# Omega Nucleic Acids (ΩNA), Ultimate Nucleic Acids for Future Technology

**DOI:** 10.3390/molecules31030523

**Published:** 2026-02-02

**Authors:** Shogo Hamada, Keiji Murayama, Yusuke Takezawa, Ryojun Toyoda, Akinori Kuzuya

**Affiliations:** 1 Department of Computer Science, School of Computing, Institute of Science Tokyo, 4259 Nagatsuta-cho, Midori-ku, Yokohama 226-8501, Kanagawa, Japan; 2 Department of Systems and Control Engineering, School of Engineering, Institute of Science Tokyo, 4259 Nagatsuta-cho, Midori-ku, Yokohama 226-8501, Kanagawa, Japan; 3 Department of Biomolecular Engineering, Graduate School of Engineering, Nagoya University, Furo-cho, Chikusa-ku, Nagoya 464-8603, Aichi, Japan; murayama@chembio.nagoya-u.ac.jp; 4 College of Engineering, Shibaura Institute of Technology, 307 Fukasaku, Minuma-ku, Saitama 337-8570, Saitama, Japan; takezawa@sic.shibaura-it.ac.jp; 5 Department of Chemistry, Graduate School of Science, Tohoku University, 6-3 Aramaki Aza-Aoba, Aoba-ku, Sendai 980-8578, Miyagi, Japan; ryojun.toyoda.a8@tohoku.ac.jp; 6 Department of Chemistry and Materials Engineering, Kansai University, 3-3-35 Yamate-cho, Suita 564-8680, Osaka, Japan

**Keywords:** nucleic acids, DNA, XNA, chemical biology, drug delivery, DNA computing, structural DNA nanotechnology, molecular robotics, molecular cybernetics

## Abstract

DNA and RNA, by focusing on their unique molecular properties, have transcended their role as carriers of genetic information in life and pioneered new application fields such as molecular robotics and molecular computing. However, as these technologies advance, the limitations inherent in natural nucleic acids and their ecosystems are increasingly becoming apparent as barriers to further application. To overcome these constraints, efforts to create artificial nucleic acids using chemical synthesis are underway and are now reaching a new stage of development. This paper proposes a concept of ultimate nucleic acid, “Omega Nucleic Acids (ΩNA),” as a thought experiment. We discuss the specifications required for this molecule, its implementable functions and approaches, and the construction of an ecosystem centered around ΩNA. By working backward from the characteristics of known natural and artificial nucleic acids, while envisioning next-generation artificial systems and applications in extreme environments, we aim to explore new approaches to nucleic acid chemistry and provide guidelines for constructing innovative artificial molecular systems.

## 1. Introduction

Over the 70 years since Watson and Crick discovered the double helix structure of DNA in 1953 [1], nucleic acid chemistry has advanced dramatically. Initially, biochemical DNA synthesis using DNA polymerase and nucleotide triphosphates (NTPs) was the mainstream approach, but the phosphoramidite DNA chemistry established by Caruthers et al. in the 1980s enabled efficient and reliable solid-phase synthesis of DNA for reasonable cost [2]. This chemistry extensively contributed to the Human Genome Project (launched in 1990) [3], which utilizes the Sanger sequencing method published in 1977 [4], and its earlier-than-planned release of a draft genome sequence in 2000. Moreover, the PCR method, published in 1983 [5], also played a major role in its widespread adoption as a technology for synthesizing the primers essential for the reaction. The above two examples of DNA chemical synthesis applications (DNA sequencing and PCR) utilize natural nucleic acids. In contrast, the antisense method, developed in 1978 [6], led to the development of various modified artificial nucleic acids. This method aims to inhibit the expression of specific genes by hybridizing a short synthetic nucleic acid strand (the antisense strand) with a sequence complementary to mRNA (the sense strand). This inhibition occurs through either blocking translation at the ribosome or degradation by RNase H. To achieve this, the antisense strand must:

1. Possess high sequence recognition ability to minimize off-target activity;
2. Form a stable double helix, preferably an A-type double helix, unlike DNA which favors B-type double helices;
3. Be recognized as a substrate for RNase H, which degrades RNA in DNA/RNA heteroduplexes.

Particularly, points 1 and 2 above are sometimes conflicting requirements, often posing significant challenges for nucleic acid chemists. Despite this, starting with the invention of PNA [7] in 1991 and BNA [8]/LNA [9] from 1997 onward, numerous excellent and highly varied artificial nucleic acids have been developed. As shown in Table 1, even listing “Nucleic Acids” found in papers, either artificial or native, abbreviated with three-letter alphabets alone occupied nearly all letters of the English alphabet within the first quarter-century. Adding artificial nucleic acids designated by abbreviations longer than three letters increases this total several-fold.

In addition to these medical applications, numerous research fields utilizing nucleic acids, such as structural DNA nanotechnology [10], DNA computing [11], and molecular robotics [12], have emerged since the 1990s through the first quarter of the 21st century. In these fields, practical environments for nucleic acid use are envisioned not only in idealized test tubes, moderately complex cells, and living organisms, but also in natural environments including soil and land/seawater. Furthermore, considering future applications in deep-sea and space exploration, environments ranging from high-temperature/high-pressure to vacuum/cryogenic conditions and high-radiation environments must also be accounted for. In this era, we believe a shift from the traditional product-oriented approach to an application-oriented approach, selecting and using the optimal synthetic nucleic acid based on the intended purpose, is essential. From this perspective, we propose a concept of ultimate nucleic acid for specific purposes, “Omega Nucleic Acids (ΩNA),” as a main R&D target for the next quarter century. Aiming to establish “ΩNA Science,” which involves selecting the optimal synthetic nucleic acid for each application and environment and freely combining them as needed, this perspective provides an overview of the outstanding synthetic nucleic acids developed to date. We also examine the requirements ΩNA must fulfill while looking ahead to both new and existing application ranges.

**Table 1:** Various “Nucleic Acids”, either artificial or native (in italic).

Acronym	Origin	Reference
ANA	Arabinonucleic Acids	[13]
BNA	Bicyclo Nucleic Acids	[8]
	Bridged Nucleic Acids	[14]
CNA	Clickable Nucleic Acids	[15]
	Constrained Nucleic Acids	[16]
	*Circulating Nucleic Acids*	[17]
DNA	*Deoxyribonucleic Acids*	[1]
ENA	Ethylene-bridged Nucleic Acids	[18]
FNA	Flexible Nucleic Acids	[19]
GNA	Glycol (Glycerol) Nucleic Acids	[20]
HNA	Hexitol Nucleic Acids	[21]
INA	Intercalating Nucleic Acids	[22]
JNA	(undefined)	-
KNA	(undefined)	-
LNA	Locked Nucleic Acids	[9]
MNA	Morpholino Nucleic Acids	[23]
NNA	Nanodiscoidal Nucleic Acids	[24]
ONA	Oxepane Nucleic Acids	[25]
PNA	Peptide Nucleic Acids	[7]
QNA	(undefined)	-
RNA	*Ribonucleic Acids*	[26]
SNA	Serinol Nucleic Acids	[27]
TNA	Threose Nucleic Acids	[28]
	Threoninol Nucleic Acids	[29]
UNA	Unlocked Nucleic Acids	[30]
VNA	*Viral Nucleic Acids*	[31]
	Virtual Nucleic Acids	[32]
WNA	W-shape Nucleic Acids	[33]
XNA	Xeno (Xenobiotic) Nucleic Acids	[34]
YNA	(undefined)	-
ZNA	Zip Nucleic Acids	[35]
	Phosphonomethylglycerol	[36]

## 2. DNA–Artificial Molecular Machine Hybrid

Intricate life activities of living organisms originate from biological molecular machines operating dynamically at the nanoscale. Key examples include kinesins, myosins, and ATP synthase. Inspired by the principles of these natural systems, scientists have designed and synthesized artificial molecular machines capable of executing mechanical tasks at the nanoscale. These artificial systems are powered by external energy input, such as light, heat, and chemical fuels, inducing conformational changes that enable various mechanical actions like switching, sliding, and rotation. Precise manipulation of nanoscale phenomena and structures represents one of the most challenging pursuits for scientists. For this purpose, the integration of stimuli-responsive artificial molecular machines with biomolecules is a promising strategy. DNA’s inherent programmability, stemming from the predictable Watson–Crick base pairing between its constituent nucleotides, allows for the precise design and self-assembly of complex nanostructures as exemplified by DNA origami techniques. Meanwhile, artificial molecular machines can be utilized to provide dynamic control over the structure and functions in response to external energy input. The combination of these two powerful classes leads to dynamic ΩNA with programmable functionality.

### 2.1. Light-Driven Molecular Machines

Chemists have developed a wide variety of artificial molecular machines. The ability of these machines to precisely control molecular conformation and motion represents a significant advance towards the sophisticated manipulation of nanostructures and their functionalities. As previously discussed, dynamic changes in molecular motion can be induced by external stimuli such as light, heat, pH, mechanical forces, and chemical fuels. Among various energy sources, photoenergy is particularly crucial for precise nanoscale manipulation [37,38,39,40,41,42,43,44,45]. Light offers a unique advantage as a non-invasive and remotely controllable stimulus for operating artificial molecular machines. Furthermore, the nature of light allows for spatiotemporal control over the stimulation, enabling localized and timed activation of molecular machines. This light-induced control is typically achieved by incorporating photo-responsive molecules into the design of molecular systems. Well-known examples include azobenzenes [46,47,48], stilbenes [49,50], diarylethenes [51,52], and spiropyrans [53,54], as well as synthetic light-driven molecular motors [45,55,56,57]. These molecules undergo reversible photoisomerization upon exposure to specific wavelengths of light, causing changes in the molecular structure. These conformational changes can then be leveraged to drive mechanical motion, such as bending, folding, ring opening and closing, and unidirectional rotation.

### 2.2. DNA Hybrid with Photoswitches

**Figure 1:**
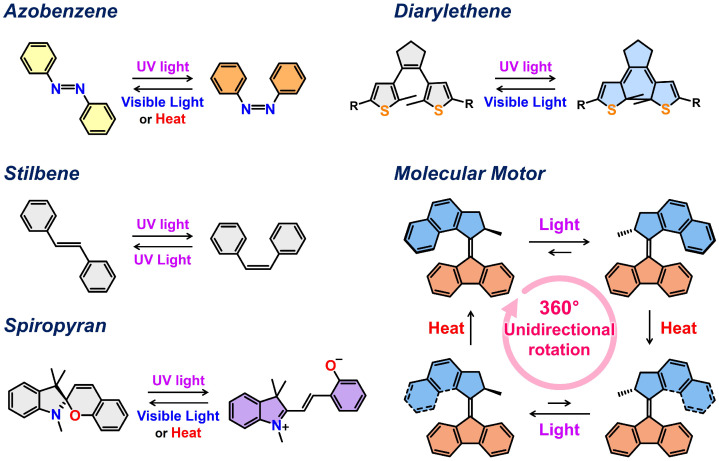
Examples of light-driven molecular machines.

**Figure 2:**
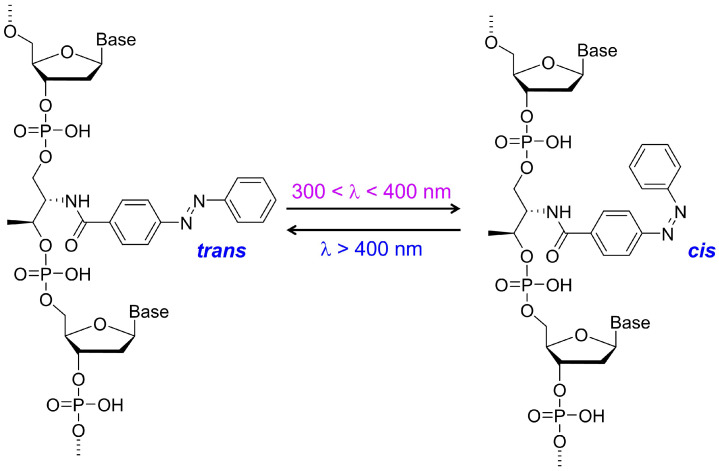
Azobenzene photoswitch dictating DNA hybridization by light irradiation.

Azobenzene and its derivatives are among the most frequently used molecular switches for photo-responsive DNA hybrids [58,59,60,61,62,63,64]. An azobenzene unit features a N=N double bond and undergoes reversible *trans*–*cis* photoisomerization upon n-π* or π-π* excitations using UV/visible light (Figure 1). This light-induced conformational change can be harnessed to drive mechanical motion in DNA-based systems. In 1999, Komiyama et al. demonstrated that DNA hybridization can be controlled by utilizing the variable structure of azobenzene in response to external light stimuli [65]. By using azobenzene-tethered DNA strands, the photo-responsive unit can be incorporated into double-stranded DNA, where hydrogen bonding between complementary nucleic acids plays a pivotal role in hybridization. In the *trans* state, the azobenzene unit is accommodated within the double helix as a result of its nonpolarity and planar structure. Meanwhile, in the *cis* state generated upon light irradiation, the azobenzene unit becomes bulkier, thereby lowering the melting temperature of the duplex, disrupting hybridization and separating the DNA duplex into single strands. This process is reversible by irradiation with light of a different wavelength, which returns the azobenzene core to the *trans* state and allows the strands to re-hybridize (Figure 2) [40,66].

**Figure 3:**
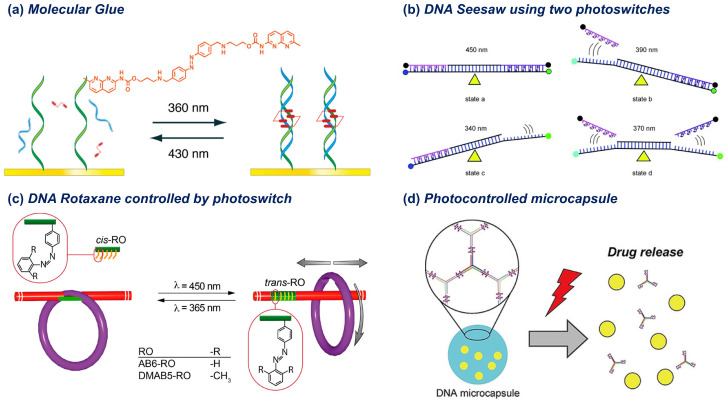
Functional DNA systems using azobenzene photoswitches. (**a**) Molecular glue. Reused from ref. [69] with the permission from ACS publications. (**b**) DNA seesaw. Reused from ref. [71] with the permission from John Wiley and Sons. (**c**) DNA rotaxane. Reused from ref. [73]. Copyright 2012 American Chemical Society. (**d**) DNA microcapsule. Reused from ref. [74] with the permission from John Wiley and Sons.

Since the pioneering work, azobenzene–DNA hybrids have been extensively applied in the photoregulation of biological phenomena and nanoscale systems. A number of comprehensive review articles cover this topic [38,40,44,67,68]. Therefore, this section will not attempt to cover all the studies but will highlight several representative works. For instance, Nakatani et al. synthesized an azobenzene derivative bearing guanine-recognizing functional groups, which acts as a photoswitchable molecular glue that controls the hybridization of mismatch DNA sequences (Figure 3a) [69,70]. In another example, Asanuma’s group reported a sophisticated DNA system where two different azobenzene-modified DNA strands are incorporated into a duplex, and the hybridization can be regulated with seesaw-like motion by selecting appropriate excitation wavelengths (Figure 3b) [71]. Tanaka et al. created a photocontrollable DNA capsule based on 3-point star motifs with azobenzene-modified sticky ends [72]. Famulok et al. developed a DNA rotaxane exhibiting macrocycle mobility controlled by photoirradiation (Figure 3c) [73]. Furthermore, photocontrollable DNA microcapsules were developed by incorporating azobenzene units into the sticky-end regions of a three-way junction motif (Figure 3d) [74]. Upon photoisomerization, this system triggers the release of encapsulated small molecules, demonstrating its potential for drug-delivery applications. With the recent trend in coacervate microdroplets produced by liquid–liquid phase separation (LLPS), this system has been exploited in other studies, and more recently, reversible photocontrol of phase transition and mechanical actions has been demonstrated by using azobenzene-based photoswitches [75,76,77]. Azobenzenetrimethylammonium bromide (azoTAB), a derivative of azobenzene, has also been employed to control the formation and dissolution of coacervate droplets by dsDNA [78]. Other classes of DNA-based systems, including molecular computers [79,80,81], DNA nanodevices [82], and molecular robots (details described in Section 5.3), have been designed by utilizing such reversible photoregulation using azobenzene and its derivatives.

DNA hybrids with other photoswitches are also intriguing. Another example of modified nucleic acids for light-controlled hybridization is spiropyran-conjugated DNA [83,84]. Incorporation of spiropyran allows photoswitching of DNA hybridization in a manner opposite to that of azobenzene. UV irradiation induces conversion from the perpendicular (closed) spiropyran form to the planar (open) merocyanine form, thereby promoting duplex formation. In contrast, visible light reverses this process by converting merocyanine back to the closed spiropyran form, which inhibits hybridization. Although applications of spiropyran in molecular computing have so far been limited, such as its use in an AND-gate probe system without DNA conjugation [85], its unique switching mechanism, complementary to azobenzene, represents a promising avenue to explore in future ΩNA designs.

Meanwhile, Irie et al. developed diarylethenes (DAEs) as photochromic switches for the first time in 1988 [51,86]. They can transform their molecular structures from the open form to the closed form and vice versa with light irradiation of different wavelengths (Figure 1). Upon the structural change from the open-ring isomer to the closed-ring isomer, the π-conjugation is expanded and the photoswitch undergoes a color change from colorless to vivid visible color. This beautiful photochromic behavior is reversible and robust even with hybridized DNA. Jäschke and the coworkers have developed DAE-modified nucleosides and the resulting photoswitchable DNAs. Spectroscopic approaches confirmed their photoswitching behaviors, demonstrating the suitability in practical applications [87,88,89]. Their design might be useful in bio-applications due to several factors: the tunability of expressed colors based on the chemical modifications, the control of fluorescence behaviors for Förster Resonance Energy Transfer (FRET) and their suitability for bioimaging with super-resolution microscopy. They have demonstrated that the photoswitch-modified dsDNA can actually regulate the in vitro transcription reactions, proving the effectiveness of DAEs in controlling biological processes.

In research on incorporating DAE into DNA, a key approach has been to link DAE to the 5-position of 2′-deoxyuridine [90]. Nucleoside-based DAEs have been developed where a purine or pyrimidine base in nucleosides becomes part of the photochromic system’s aryl ring. This is expected to allow light to directly and powerfully influence the structure and function of the nucleoside. DAE-modified nucleosides can be introduced into a DNA strand via solid-phase synthesis using automated phosphoramidite chemistry, which offers higher yields and site-specific introduction compared to the previous Suzuki coupling method, which had low yields and made multiple DAE insertions difficult.

To give examples of functional DAE-DNA hybrids, a highly efficient photoswitch based on FRET was constructed by incorporating a doubly methylated dU-Me-PhtBu as a fluorescent donor and tricyclic cytidine (tC) as an acceptor into DNA. FRET is efficient when the DAE is in its open state (ON) but is inhibited when the DAE is in its closed state (OFF), where no fluorescence is emitted. This system operates in both liquid and solid phases, maintaining excellent ON/OFF contrast and fatigue resistance over 100 cycles. Orientation-dependent energy transfer is also observed due to the chromophores being fixed within the DNA double helix. This design significantly simplifies the system by combining the donor and modulator properties into a single entity [91]. Another remarkable study is light-control of DNA-binding helical peptides [92]. A molecular system was developed to control the α-helical structure and DNA binding affinity of a helical peptide, employing DAE as a cross-linker. QCM (Quartz Crystal Microbalance) analysis confirmed that peptides with the open-ring DAE have a higher DNA binding affinity than those with the closed-ring form. Light irradiation during QCM measurement demonstrated that the light-controlled DNA–peptide interaction could be modulated in real-time.

These studies show that DAE’s photochromic properties are maintained within a DNA environment and exhibit sequence-independent switching behavior. This provides important guidelines for designing more efficient and functional DNA photoswitches for bionanotechnology and synthetic biology.

### 2.3. DNA Hybrid with Light-Driven Rotary Molecular Motors

**Figure 4:**
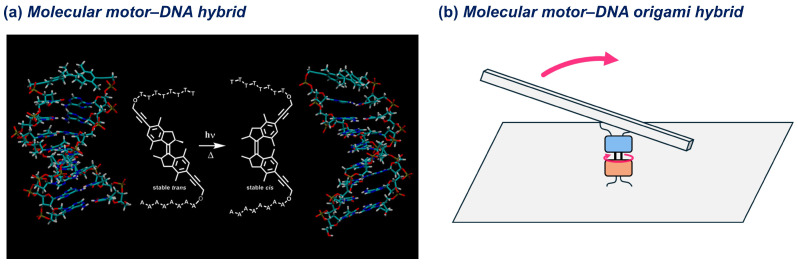
Hybrids between molecular motor and DNA. (**a**) Photoswitching DNA hybridization. Adapted from [94], CC-BY-NC-ND. (**b**) Rotary DNA nanostructure driven by an artificial molecular motor [95].

Another masterpiece of molecular machines is the artificial rotary molecular motor, first developed by Feringa and his coworkers [93]. These motors perform continuous, unidirectional 360-degree rotation through a sequence of photochemical and thermal processes. Typical molecular structures of these motors are illustrated in Figure 1. Upon light exposure, photoisomerization around the double-bond axle is induced, leading to a metastable state. From this strained state, the structure relaxes to a more stable conformation via thermal helix inversion. The thermal step is energetically downhill and critically prohibits random reversal while rectifying thermal fluctuations, thereby ensuring unidirectional movement. These two processes complete a unidirectional 180-degree rotation of the molecular motor. Continuous 360-degree rotation is achieved by repeating these light and heat input cycles. Light-driven molecular motors operate in diverse environments, including organic solvents, aqueous media, liquid crystals, on surfaces, and within solid materials, and their unique capability makes them promising candidates for future nanoscale mechanical manipulation in DNA-based nanomaterials. In this context, several pioneering examples of hybridization of artificial molecular motors and DNA strands have been reported. For example, Feringa group demonstrated photocontrol of DNA hybridization using a molecular motor (Figure 4a) [94]. A multi-state photoswitchable DNA hairpin was developed by introducing a first-generation molecular motor into a self-complementary DNA strand. They showed that the molecular motor undergoes photoisomerization and thermal helix inversion even within the hybrid system. This light-driven rotational motion induced large structural changes and destabilized the DNA duplex upon *trans*–*cis* isomerization. The conjugation of molecular motors and DNA materials has advanced further. Recently, Helmi et al. reported a rotary DNA nanostructure powered by a synthetic molecular motor (Figure 4b) [95]. A second-generation molecular motor was integrated into a robust DNA origami nanostructure, enabling nanometer-precision positioning of the motor and connecting the rotor arm to the anchoring surface. Upon UV irradiation, the torque generated by the unidirectional rotation of the tiny motor was transferred to the DNA rotor arm. As a result, a larger scale motion was induced, which was successfully observed by single-molecule imaging technique. This study is reminiscent of the work by Kosuri et al., reporting the rotational motion of a DNA nanostructure with the use of a motor protein [96]; however, the artificial motor provides the active control of the rotational dynamics with light. This achievement introduced a design principle for nanoscale robotic devices.

The above-mentioned and related studies highlight the huge potential of combining artificial molecular machines with DNA-based materials, and open a new avenue for dynamic functions of ΩNA. However, it should be noted that these molecular machines are often driven by light in the UV region, which can damage or deteriorate biomolecular systems, e.g., inhibiting transcription. To further advance this research field, it will be required to develop molecular machines capable of responding to visible to near infrared light with high sensitivity. Also, photoluminescence properties would be beneficial for tracking their motion as a single-molecular level in biosystems.

## 3. Xeno-Nucleic Acids (XNAs)

**Figure 5:**
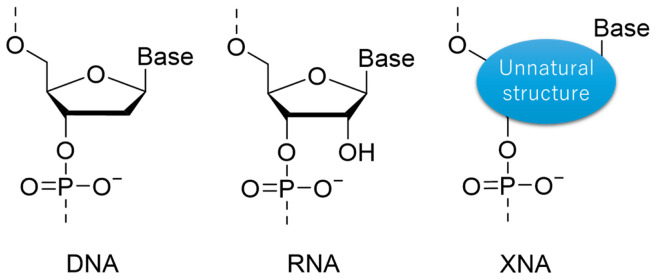
DNA, RNA, and XNA.

Chemical modifications on DNA and RNA oligomers enable expansion of supramolecular function, promising various applications. Phosphoramidite chemistry is one of the most reliable methodologies that produces a variety of modifications on the nucleobase, phosphate, and sugar scaffold. Among these modifications, artificial nucleic acids that have unnatural scaffolds instead of a D-ribose-based structure are referred to as xeno-nucleic acids (XNAs). Chaput and Herdewijn discussed the needs for re-definition and re-classification of XNAs [34], which has not yet been fully codified. We herein describe XNA as a synthetic genetic polymer based on modified scaffolds other than 2’-deoxyribose and ribose, and distinct from chemically modified DNA that contains modification only on nucleobase and on phosphodiester linkage (Figure 5). We further classify XNAs into two groups: cyclic scaffolds [97] and acyclic scaffolds [98]. To ensure the formation of duplexes and multiplexes as with DNA and RNA, most of cyclic XNA scaffolds have been basically designed to be similar to D-deoxyribose and D-ribose. In some cases, such cyclic XNAs can be a substrate of an enzymatic reaction due to the resemblance of the scaffold structure to the natural system. In contrast, acyclic scaffolds have high enzymatic durability and orthogonality to natural enzymes due to largely different structures compared to those of natural cyclic sugar-based scaffolds. Furthermore, acyclic XNAs composed of a non-sugar-based backbone avoid the issue of depurination that readily occurs in DNA/RNA under low pH conditions. In addition, a unique hybridization property hugely different from DNA and RNA can be achievable if it was properly designed. However, a reasonable design of the acyclic scaffold is still difficult. In this section, we summarize the hybridization properties and applications of representative XNAs.

### 3.1. Design and Hybridization Properties of Cyclic XNAs

#### 3.1.1. $2^{\prime }$-*O*-alkyl RNA

**Figure 6:**
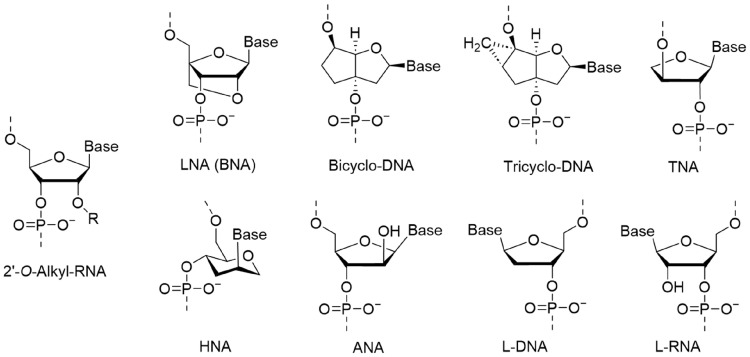
Chemical structures of cyclic XNAs.

To our knowledge, the first reported XNA with cyclic scaffold is $2^{\prime }$-*O*-alkyl RNAs (Figure 6) [99]. These modifications increase the chemical stability of the oligomer due to the protection from hydrolysis of phosphate because 2′-hydroxyl group is absent. Nuclease-stability is also facilitated. The $2^{\prime }$-*O*-alkyl substitution basically does not affect the hybridization ability; however, substitution with large molecules tends to decrease the thermal stability of the duplex [100]. Among various modifications, $2^{\prime }$-*O*-methoxy-ethyl RNA ($2^{\prime }$-MOE) is often used in nucleic acid medicines that are clinically approved [101,102]. The $2^{\prime }$-*O*-Alkyl RNA has high structural compatibility with RNA, which does not largely alter the native hybridization properties and duplex structures, resulting in various biological applications.

#### 3.1.2. LNA (BNA) Family

One of the most widely used cyclic XNA for biological applications is locked nucleic acid (LNA) [9] which is also referred to as bridged nucleic acid (BNA) (Figure 6) [8]. LNA has a backbone structure in which the $2^{\prime }$-oxygen and the $4^{\prime }$-carbon are covalently connected. DNA and RNA suffered from entropy loss for duplex formation due to conformational transition between C$2^{\prime }$-endo and C3*’*-endo puckering of ribose. In contrast, LNA can form a highly stable duplex with complementary DNA, RNA and LNA, since single-stranded LNA is pre-organized to adopt the A-type duplex because of the fixed C$3^{\prime }$-endo conformation [103]. As an example, fully modified LNA oligomer, the sequence composed of only LNA without DNA, extremely increased its *T*_m_ by 35 °C in 9-mer sequence [104]. In addition, LNA modification also increases durability against enzymatic digestion [105]. As a family of LNA, a lot of derivatives have been reported for further functionalization [106,107]. This strategy was expanded to further rigid XNA scaffolds such as bicyclo-DNA and tricyclo-DNA (Figure 6) [108,109]. The tricyclo-DNA forms homo-duplex and hetero-duplexes with complementary DNA and RNA with obviously increased thermal stability [110].

#### 3.1.3. Other Cyclic XNAs

Eschenmoser et al. reported α-Threofuranosyl-($3^{\prime }$→$2^{\prime }$) Oligonucleotide (TNA) as a candidate for XNA in pre-RNA world [28]. TNA has tetrose sugar, α-(L)-threose, having only two carbons between phosphates, whereas DNA and RNA have three carbons (Figure 6). Despite such structural difference from natural nucleic acids, TNA forms duplexes with complementary TNA, DNA and RNA. However, the thermal stabilities of these duplexes were even lower than those of DNA and RNA.

Hexitol nucleic acid (HNA) was reported by Herdewijn group as anti-viral reagent at first (Figure 6) [21]. HNA oligomers constructed of 6-membered ring $1^{\prime }$,$5^{\prime }$-anhydrohexitol building blocks also hybridized with complementary HNA, DNA and RNA [111,112]. In the case of HNA, hetero-duplexes with DNA and RNA are thermally stabilized compared to duplexes of DNA and RNA. This stabilization would be attributed to the structure of HNA building unit accommodating A-form duplex. Damha’s group developed oligomer of arabinonucleic acid (ANA) (Figure 6) [13]. Fully ANA strand does not form stable homo-duplexes [113], whereas the ANA forms a thermally stable duplex with complementary RNA strand [13]. ANA and RNA monomers can be synthesized from a common intermediate, indicating the potential that the ANA can be a genetic polymer as with RNA in the early Earth [114]. L-DNA and L-RNA are mirror images of natural D-DNA and D-RNA (Figure 6). The chemical and hybridization properties of these L-oligonucleotides are completely the same as natural D-oligonucleotides. However, interestingly, L-DNA cannot hybridize with D-DNA even when the sequence is complementary with each other, due to the helical mismatch [115]. It is also worth noting that L-DNA and L-RNA are hardly degraded by nucleases in nature. Such orthogonality against natural systems is a strong advantage of these L-oligonucleotide-based XNAs.

### 3.2. Applications of Cyclic XNAs

**Figure 7:**
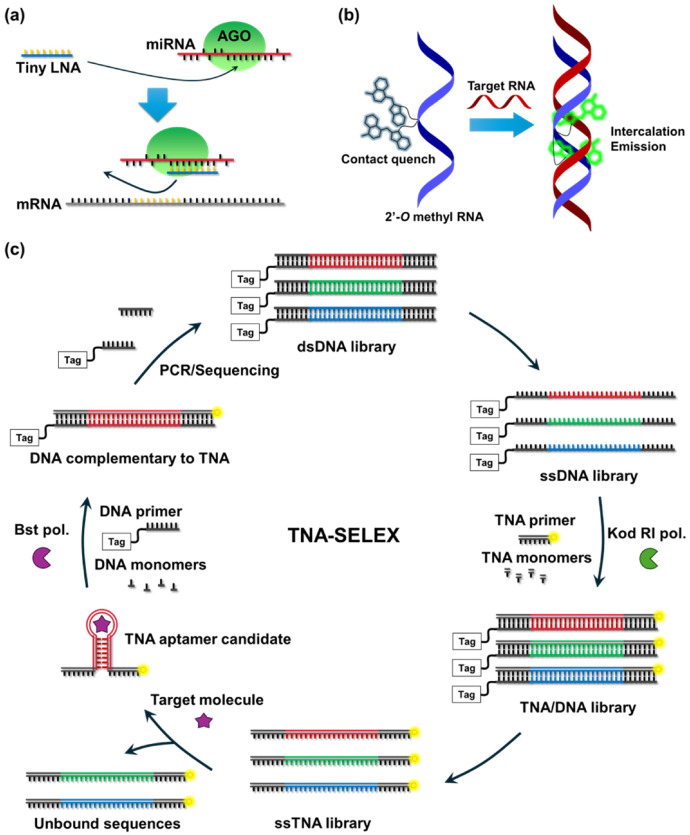
Schematics of (**a**) tiny LNA as miRNA-targeting medicines [122], (**b**) echo probe visualizing target RNA in cell [127], and (**c**) TNA-based SELEX system for TNA aptamer [134].

Cyclic XNAs were valuable as a scaffold of tools for oligonucleotide therapeutics [116]. Because cyclic XNAs have a sufficient binding ability to complementary RNAs and high enzymatic durability in living cells, cyclic XNA-based RNA-targeting medicines should be highly useful. As an example, siRNA containing $2^{\prime }$-*O*-methyl RNA substitution at specific positions of the guide chain reduced off-target effects of RNAi while maintaining sufficient activity against the target sequence [117]. The combination of $2^{\prime }$-modified RNA and phosphorothioate modification is commonly used in various oligonucleotide therapy applications [118,119,120]. For the practical use of RNA-targeting cross-linker, $2^{\prime }$-*O*-methyl RNA was employed as the main scaffold [121]. The strong binding affinity of LNA has provided a new strategy for clinical applications. An 8-mer LNA oligonucleotide (tiny LNA) was designed as an antisense nucleic acid for miRNAs [122]. The tiny LNA was designed to have a sequence complementary to the seed region of target miRNAs, enabling simultaneous inhibition of miRNA family that share the same seed region (Figure 7a). Both in cell experiments and in animal experiments, the tiny LNA successfully induced miRNA silencing activity with little or no off-targeting [122]. Nucleic acid aptamers are valuable biological tools that can selectively bind to the target biomolecules. L-RNA aptamer, also referred to as spiegelmer, was discovered using the mirror target, and confirmed the binding ability for the original target. L-RNA aptamer has remarkably high stability even in the human serum where conventional D-RNA is easily degraded [123].

Cyclic XNAs are also valuable as a scaffold of nucleic acid probes. A 2*’*-*O*-alkyl RNA scaffold was introduced into the molecular beacon system [124,125] and echo probe system [126,127] to realize RNA imaging in a living cell (Figure 7b). Significantly high affinity for RNA allows LNA to be used as northern blot analysis of miRNAs [128]. This LNA-based probe can also discriminate between single and double mismatches. This methodology demonstrated highly sensitive detection of target miRNA compared to conventional DNA-based methods.

Chaput et al. reported template-directed synthesis of TNA on DNA template and of DNA on TNA template using commercial polymerase and an engineered TNA polymerase, which was successfully applied to the selection of TNA aptamer (Figure 7c) [129,130,131,132,133,134]. TNA-based aptamer is biologically stable compared to conventional DNA-based aptamer that is easily degraded by nucleases. A similar strategy was performed to HNA, successfully obtained HNA aptamer by multi-round SELEX system [135]. Holliger’s group successfully developed polymerase evolution system and obtained engineered XNA polymerases. This methodology enables the evolution of XNA, which realized a selection of XNA-based aptamer [136]. Moreover, they also achieve a selection of XNAzyme that can cleave oligonucleotides in sequence specific manner [137]. XNAzymes composed of ANA, HNA, $2^{\prime }$-fluoro-arabinonucleic acids (FANA), and cyclohexene nucleic acids (CeNA), were successfully selected from random XNA oligomer pools. These technologies enable the discovery of catalysts of diverse XNA scaffolds that do not exist in nature. The evolution of such catalytic activity provides important insights into the definition of chemical boundary conditions for the emergence of life.

### 3.3. Design and Hybridization Properties of Acyclic XNAs

#### 3.3.1. PNA

**Figure 8:**
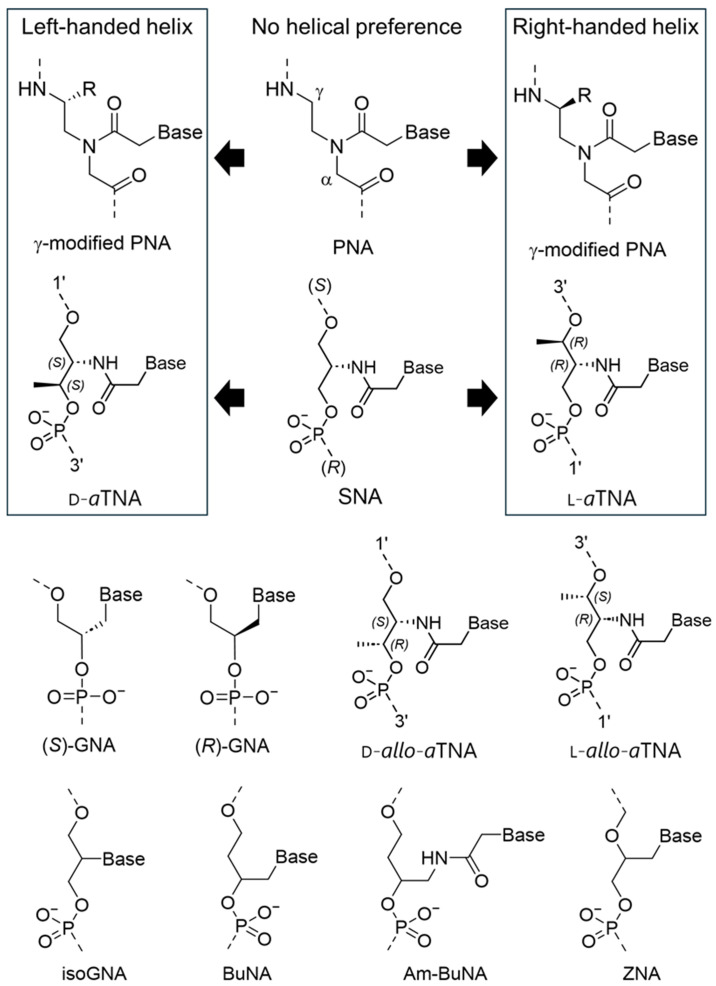
Chemical structures of acyclic XNAs.

The first acyclic XNA with sufficient hybridization ability is peptide nucleic acid (PNA) composed of a peptide-based scaffold (Figure 8) [7,138]. The peptide-based charge neutral backbone is free from electrostatic repulsion upon duplex formation with complementary strand, although the neutral backbone has two drawbacks: low water solubility and limitation on the synthesis of sequences. Presumably, large entropy loss of flexible acyclic scaffold on duplex formation was compensated by the liberation of repulsion between negatively charged phosphate anions. As a result, PNA can form thermally stable duplexes with DNA and RNA. Pentadecamer of PNA/DNA duplex showed 16 °C higher *T*_m_ than DNA/DNA duplex with the same sequence, as an example [139]. In addition, PNA/PNA homo-duplexes are also highly stable compared to natural DNA and RNA. Such peptide-based structure is largely different from natural DNA and RNA, resulting in high durability against nucleases. Interestingly, poly pyrimidine PNA strands are known to form extremely stable triplexes with DNA poly purine strand [140,141]. Another characteristic feature of PNA is the achiral backbone due to the absence of the chiral center on the main chain structure, resulting in no helical preference. Chiral modification on a or g position of the PNA scaffold induces helicity: right-handed PNA enables selective binding to natural right-handed D-D(R)NA, not to left-handed L-D(R)NA. In contrast, left-handed PNA has high affinity for L-D(R)NA and is orthogonal to D-D(R)NA [142,143,144,145]. Interestingly, cyclic PNA structures have also been investigated to further enhance the hybridization property [146,147,148,149].

#### 3.3.2. GNA

Zhang and Meggers et al. reported glycol nucleic acid (GNA), sometimes referred to as glycerol nucleic acid, as the simplest scaffold of acyclic XNAs (Figure 8) [20,150]. The structural design of GNA was based on TNA. Until GNA was reported, it was believed that acyclic XNAs with phosphodiester bonding had difficulty forming stable duplexes [151,152,153]. Nevertheless, GNA forms GNA/GNA homo-duplexes via Watson–Crick base pairing with significantly high *T*_m_ compared to natural nucleic acids. The *T*_m_ of 15-mer GNA/GNA duplex was 71 °C, which was much higher than the 47 °C, *T*_m_ of the DNA duplex of the same sequence under the same conditions. Facile synthesis of GNA is also an advantage for applications. The phosphoramidite monomers of GNA are easily derived from glycidol. The design of the chemical structure of GNA was expanded to various new acyclic XNAs such as isoGNA [154], BuNA [155], Am-BuNA [156], and ZNA [36]. Interestingly, the chimeric strand containing GNA and DNA significantly reduced the duplex formation ability [150]. Chirality of the scaffold of GNA significantly affects the hybridization ability. (*S*)-GNA oligomer and complementary sequence of (*R*)-GNA does not form a duplex, probably due to helical mismatch [29,36]. Only (*S*)-GNA recognizes RNA even though the sequences should not be composed of G and C.

#### 3.3.3. *a*TNA/SNA

Our group developed acyclic threoninol nucleic acids (*a*TNAs) with four different chirality: D-*a*TNA, L-*a*TNA, D-*allo*-*a*TNA, and L-*allo*-*a*TNA (Figure 8) [29,157,158,159]. To discriminate from α-Threofuranosyl-($3^{\prime }$→$2^{\prime }$) Oligonucleotide (TNA), we added “*a*” in the abbreviation. In addition, since there are two chiral carbons in the scaffold of *a*TNA, D,L label was adopted to indicate the chirality. D-*a*TNA oligomer and L-*a*TNA oligomer form a stable homo-duplex with complementary D-*a*TNA and L-*a*TNA, respectively, showing considerably higher melting temperature compared to DNA homo-duplexes [29,157]. However, the hetero-duplex of D-*a*TNA and L-*a*TNA was not observed due to helical mismatch. We found that D-*a*TNA does not form hetero-duplexes with either D-DNA or D-RNA, whereas L-*a*TNA forms hetero-duplexes with these natural nucleic acids [98,160]. Thus, we concluded that D-*a*TNA and L-*a*TNA prefer a left-handed helix and right-handed helix, respectively. As a result, helical orthogonality was achieved. Multiplex formation of *a*TNA was also investigated by Gothelf’s group. L-*a*TNA poly T was found to form an extremely stable triplex with DNA poly A and RNA poly A [161]. An i-motif formation [162] and a G-quadruplex formation [163] were also suggested. The *allo*-*a*TNAs showed very weak hybridization property compared to *a*TNAs, probably due to wrong configuration of methyl group [158]. Deletion of methyl group from threoninol resulted in achiral serinol backbone. We also developed serinol nucleic acid (SNA) using serinol unit as a scaffold (Figure 8) [27]. Helical preference of SNA scaffold was absent due to achiral scaffold. As a result, SNA can form a duplex with both right-handed and left-handed nucleic acids. SNA forms stable homo-duplexes and hetero-duplexes with complementary DNA and RNA.

### 3.4. Applications of Acyclic XNAs

**Figure 9:**
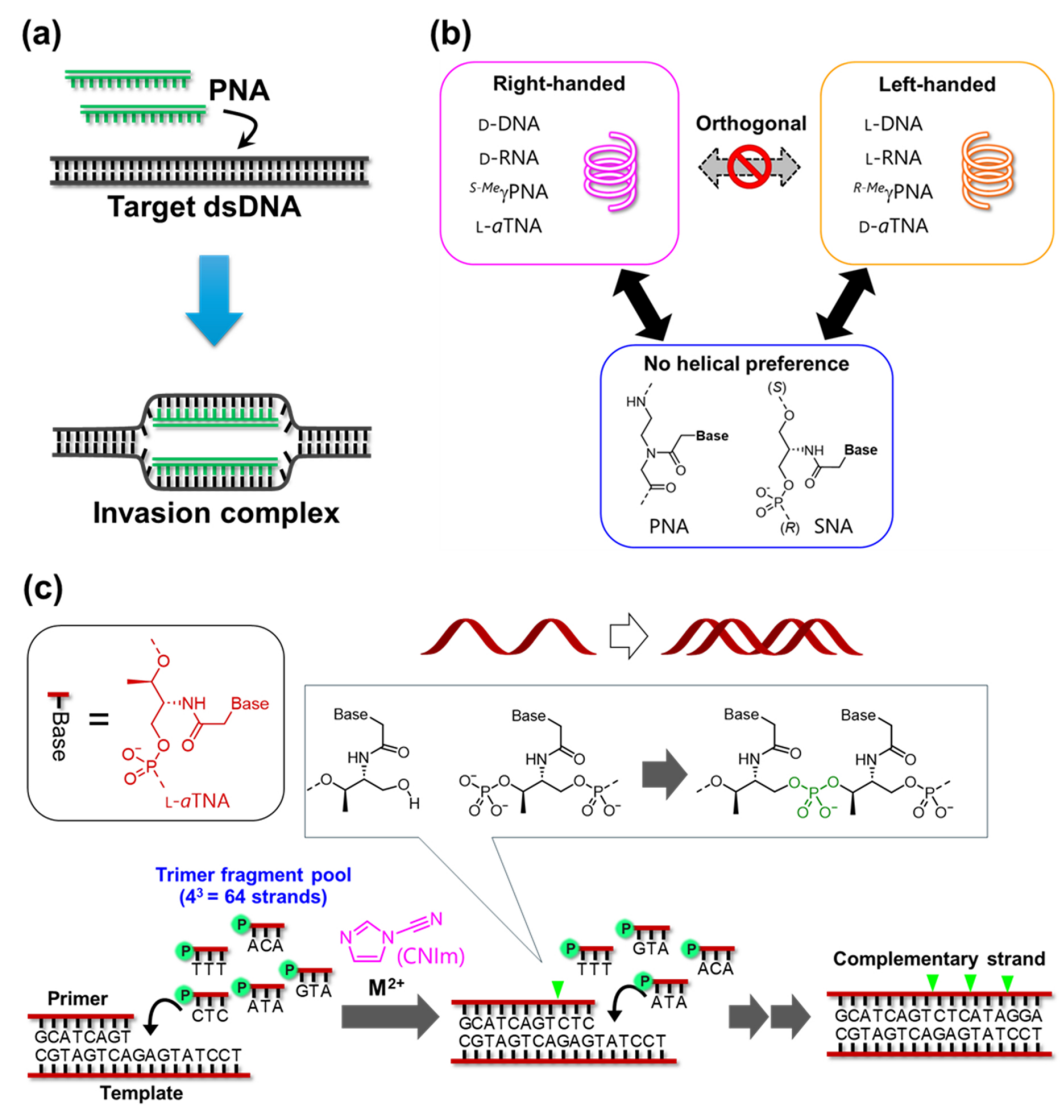
Schematics of (**a**) strand invasion of PNA into DNA duplex [164], (**b**) helical property and cross-talk ability among acyclic XNAs (Copyright 2023 Chemical Society of Japan) [190], and (**c**) non-enzymatic primer extension of L-*a*TNA [199].

Absence of electrostatic repulsion between PNA and complementary DNA facilitates invasive hybridization of PNA strands into dsDNA: the PNA strand is inserted into the target region of DNA and forms a PNA/DNA hetero-duplex through a partial dissociation of the DNA duplex (Figure 9a) [164,165]. The invasion technique promises a detection of dsDNA in situ and a genome editing technology. PNA hybridization to an mRNA sterically blocks the splicing or translation [166,167]. Splicing correction using this strategy was demonstrated in a transgenic mouse [168]. a-modified PNA was also used to effective inhibition of EGFR expression in a mouse model [169]. Triplex-forming PNA oligomer targeting dsRNA is effective to suppress the translation of mRNAs [170]. L-*a*TNA- and SNA-based therapeutics have huge advantages due to high nuclease resistance and sufficient hybridization ability for RNA. SNA was successfully applied to antisense oligonucleotide (ASO) for splice-modulation [171]. The phosphorothioate-modified gapmer ASO with SNA targeting sodium glucose co-transporter 2 (SGLT2) showed early increase in renal uptake, reduction in renal SGLT2 expression, and subsequent glucosuria. Importantly, SGLT2-SNA-ASO did not cause severe liver damage [172,173]. Anti-miRNA oligonucleotide (AMO) composed of fully SNA showed strong nuclease resistance and effectively suppressed miRNA activity [174]. The experiments in a mouse model of cystic kidney disease and human ADPKD cells revealed that anti–miR-21-SNA effectively prevented cyst growth in vivo and in vitro, indicating a high potential as a therapeutic strategy. Recently, a new gene-suppressing method was achieved using a short L-*a*TNA oligomer, a Staple oligomer. The Staple oligomer hybridizes specifically to a target mRNA and artificially induces an RNA G-quadruplex, resulting in the effective suppression of the target protein’s translation. This technology provides new insights into gene therapy after RNAi and antisense technologies [175].

PNA has been utilized as hybridization-based fluorescent probes such as FIT-probes, which achieved RNA imaging with low background noise in solution and in cells [176,177,178,179]. Thiazole-based dye incorporated into PNA strand is quenched in single stranded state due to free rotation of the methyn position, whereas the hybridization with target RNA facilitates the intercalation of fluorophore into base pairs, enhancing fluorescent emission. A detection of dsDNA was demonstrated using triplex-forming bis-PNA openers [180]. PNA is also effective for use as various types of probes targeting not only nucleic acids but also protein [181]. Molecular beacon composed of a fully SNA scaffold showed remarkably high sensitivity, high affinity for target RNA, and sufficiently high enzymatic durability [182]. The strong base pairing of the stem duplex induced highly effective quenching in the absence of target due to the suppressing of the breathing effect, resulting in higher sensitivity than conventional DNA-based molecular beacons. Such strong base pairing property of acyclic XNAs was also beneficial for the multiplexed labeling system [183]. L-*a*TNA modified with perylene-modified uracil established a quencher-free linear probe that discriminates single base mismatch with high sensitivity [184].

Incorporation of 8-pyrenylvinyl adenine (^PV^A) into SNA strand enabled photoregulation of duplex formation and dissociation with complementary RNA [41]. Two ^PV^A residues in SNA strand undergo [2+2] photocycloaddition reaction upon irradiation with blue light, inducing structural distortion dissociating the duplex into single strands. This reaction is reversed by the irradiation with Vis light. This technology was expanded to 8-naphthylvinyladenine [185] and 8- pyrenylvinyl guanine [186]. Achiral property of PNA enables hybridization with both right-handed and left-handed nucleic acids (Figure 9b). This feature can be applied to heterochiral circuits that realize signal transduction between D-DNA and L-DNA via achiral PNA as an interface using the toehold-mediated strand displacement method [187,188]. The signal transduction between right-handed PNA and left-handed-PNA is also possible using achiral PNA [145]. SNA also has achiral property, which forms a duplex with nucleic acids regardless of the helicity (Figure 9b). Hybridization Chain Reaction (HCR) was designed using D-*a*TNA and L-*a*TNA [189,190]. The *a*TNA hairpins with a short 7 base-pair stem achieved clear ON–OFF control of the HCR circuit and the D-*a*TNA circuit and the L-*a*TNA circuit were orthogonal of each other. Using SNA as an interface, D-*a*TNA HCR circuit, orthogonal to the natural system, was successfully activated by the target RNA. This method has potential for low-noise visualization of RNA in vivo and the FISH method. A membrane-penetrating molecular device was achieved using the triplex of poly T L-*a*TNA modified with cholesterol and poly A DNA [191]. This device was applied to a reversible photo-triggered signal transduction system functioning on giant unilamellar vesicles. This system was made possible precisely because it utilizes L-*a*TNA, an acyclic XNA that is relatively easy to synthesize. Four-helix junctions with left-handed (R)-GNA homoduplex and right-handed (S)-GNA homoduplex were achieved [192]. High stability of GNA homo-duplexes resulted in largely higher thermal stability for 4-helix junction structures compared to conventional DNA-based structures. *a*TNA has also been applied to the construction of thermally stable nanostructures such as four-way junction, 3D cube, and pyramid structures [193,194].

Liu’s group demonstrated sequential DNA-templated chemical ligation of PNA aldehyde using reductive amination reactions, generating PNA-based polymer from short PNA fragments [195]. Side-chain modification on PNA fragments was acceptable for this reaction [196]. They also developed the translation, selection, and amplification system for PNA using DNA as a template, providing an experimental foundation for PNA evolution [197]. Moreover, a complete cycle of translation, coding sequence replication, template regeneration and re-translation was achieved by DNA-templated translation system, promising in vitro selection [198]. Non-enzymatic template-directed synthesis of L-*a*TNA was achieved via chemical ligation mediated by N-cyanoimidazole and divalent metal cations (Figure 9c) [199]. A pseudo primer extension reaction using a pool of random L-*a*TNA trimers as substrates generated 9-mer-extended L-*a*TNA product on the L-*a*TNA template. The efficiency was further improved by optimization of the reaction conditions, resulting in as long as 21-mer elongation with 60 % yield within 2 h [200]. This technique was further expanded to “reverse transcription” from RNA to L-*a*TNA: template-directed synthesis of L-*a*TNA was possible on the RNA template [201]. These L-*a*TNA-based synthetic technologies for “artificial central dogma” would pave the way for applications in XNA-based in vitro selection, the creation of artificial life, and nanotechnologies. Sequencing project for *a*TNA and SNA is undergoing. Winssinger’s group developed the non-enzymatic primer extension system for PNA and applied it to sequencing of XNA strands [202]. The activated 4-mer PNAs were used as ingredients and ligated on template XNAs to produce a complementary strand. This technology demonstrated a sequencing of L-DNA, LNA, and PNA.

## 4. Unnatural Base Pairs (UBPs)

One of the alternative approaches to creating unnatural nucleic acid molecules involves replacing the natural Watson–Crick base pairs with their artificial analogs [203,204,205,206,207]. The introduction of unnatural base pairs (UBPs) into nucleic acids theoretically expands the information capacity encoded in base sequences. For example, when artificial **X**–**Y** base pairs are used in addition to the canonical A–T and G–C base pairs, the number of possible base sequences increases from $4^{n}$ to $6^{n}$ (where *n* is the length of the oligonucleotide). From this perspective, various UBPs have been developed, primarily aimed at expanding the genetic alphabet. Typically, UBPs are designed to (i) form base pairs selectively, thereby ensuring sequence-specific hybridization; (ii) exhibit orthogonality to natural Watson–Crick base pairs to prevent information cross-talk; and (iii) closely match the size and shape of natural bases to maintain the structural integrity of nucleic acid duplexes. In particular, artificial base pairs that can be recognized by enzymes such as DNA polymerases have attracted significant attention, owing to their applications in PCR amplification, SELEX (Systematic Evolution of Ligands by EXponential enrichment), and incorporation of unnatural amino acids into proteins. To date, a wide range of artificial base pairs have been developed, including those with hydrogen-bonding patterns different from natural base pairs, hydrophobic base pairs that form without hydrogen bonding, and metal-mediated base pairs that are formed through coordination bonds in the presence of specific metal ions. Nevertheless, their potential applications in the field of DNA nanotechnology still leave considerable room for exploration. UBPs that expand the sequence repertoire and exhibit orthogonality to natural nucleic acids are anticipated not only to enhance the programmability of DNA nanostructures but also to open up new possibilities for DNA computing. In this chapter, the representative examples of such UBPs are reviewed, and the future perspectives are discussed.

### 4.1. Hydrogen-Bonded Artificial Base Pairs

**Figure 10:**
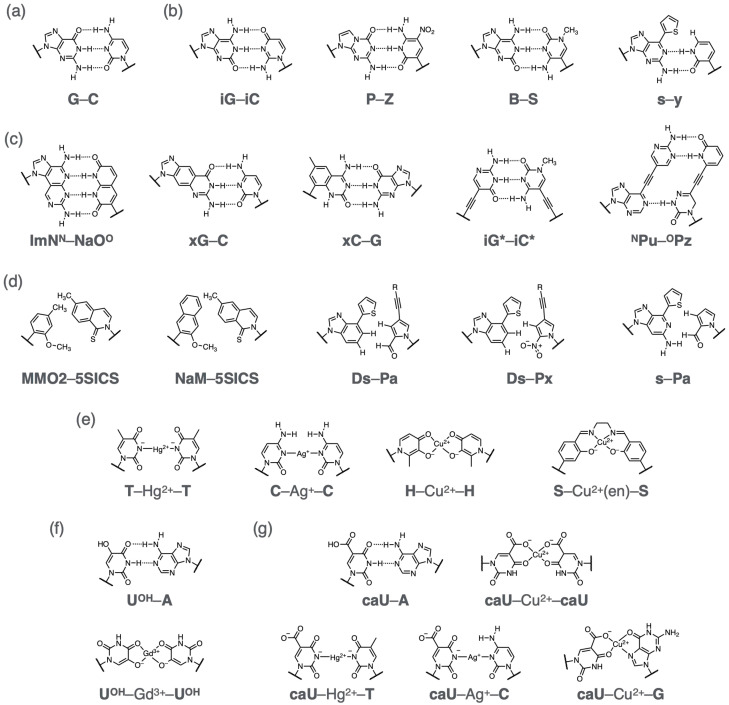
Chemical structures of unnatural base pairs (UBPs). (**a**) A hydrogen-bonded natural nucleobase pair (G–C). (**b**) Representative examples of hydrogen-bonded unnatural base pairs. (**c**) Representative examples of size-extended hydrogen-bonded base pairs. (**d**) Representative examples of hydrophobic unnatural base pairs. (**e**) Representative examples of metal-mediated unnatural base pairs. (**f**) Hydrogen-bonded and metal-mediated base pairing of 5-hydroxyuracil ($U^{\mathrm{OH}}$) bases. (**g**) Hydrogen-bonded and metal-mediated base pairing of 5-carboxyuracil (**caU**) bases.

Natural DNA (RNA) molecules hybridize with their complementary strands via hydrogen-bonded A–T (A–U) and G–C base pairing (Figure 10a). One of the earliest strategies for designing unnatural base pairs (UBPs) is to rearrange the hydrogen-bonding pattern while retaining the overall shape of canonical base pairs (Figure 10b). A pioneering example is the base pair formed of isoguanine (**iG**) and isocytosine (**iC**), in which the amino and carbonyl groups of the natural bases G and C are interchanged [208,209]. These bases can form a hydrogen-bonded **iG**–**iC** base pair with a donor–acceptor pattern distinct from that of G–C. The **iG**–**iC** base pair was shown to be accommodated by DNA and RNA polymerases. However, it faced issues such as reduced selectivity due to the keto–enol tautomerization of the **iG** base and the chemical instability of the **iC** base, which prompted the development of improved version of UBPs. For instance, Benner’s group created the **P**–**Z** base pair, where the **P** base was designed to prevent tautomerization, and the **Z** base was chemically stabilized by introducing a nitro group [210]. Other hydrogen-bonded UBPs, such as the **B**–**S** pair, have also been synthesized [211]. To enhance the orthogonality of hydrogen-bonded UBPs to natural nucleobase pairs, Hirao’s group introduced bulky substituents into artificial nucleobases. For example, the **s** base was designed to pair selectively with a relatively small **y** base via two hydrogen bonds, forming an **s**–**y** pair [212]. Although the **s** base is structurally capable of forming two hydrogen bonds with the natural T base, the formation of the **s**–T base pair is sterically hindered by the thiophene moiety at the 6-position, thereby improving the fidelity of base pairing during enzymatic replication. It should be noted that the presence of proton acceptors on the minor groove side, such as the 2-keto group of T and C and the N3 atom of A and G, plays an important role in substrate recognition by polymerases. These mechanistic insights have also been incorporated into the molecular design of UBPs capable of functioning in enzymatic amplification and transcription processes.

Other types of hydrogen-bonded UBPs have been designed by sterically expanding purine and pyrimidine scaffolds (Figure 10c). Minakawa and Matsuda et al. developed a series of UBPs formed via four hydrogen bonds [213]. Specifically, imidazo[5’,4’:4,5]pyrido[2,3-*d*]pyrimidine derivatives were designed as extended purine analogs, while 1,8-naphthyridines served as extended pyrimidine analogs, enabling the formation of an additional hydrogen bond between complementary bases. For example, the ${\mathrm{ImN}}^{N}$–${\mathrm{NaO}}^{O}$ base pair exhibited high pairing selectivity and was successfully replicated by PCR. Another approach involves expanding the nucleobase size while retaining Watson–Crick-type hydrogen-bonding patterns [214]. Kool’s group developed size-extended UBPs by fusing a benzene ring to purine or pyrimidine scaffolds [215,216]. The resulting nucleobases (**xA**, **xT**, **xG**, and **xC**) pair with their natural counterparts to form **xA**–T, **xT**–A, **xG**–C, and **xC**–G base pairs, respectively. These base pairs are approximately 2.4 Å wider than canonical base pairs, resulting in a broader duplex architecture termed xDNA. This system represents an eight-letter nucleic acid composed of four natural and four unnatural nucleobases, potentially enhancing information storage capacity. The development of xDNA demonstrated that the geometry of DNA analogs is not inherently constrained to that of the natural duplex structure. Due to the enhanced base-stacking interactions between the enlarged UBPs, xDNA is expected to exhibit increased thermal stability and rigidity, making it a promising platform for DNA nanotechnology applications. Similarly, other size-extended UBPs have been reported, including yDNA, in which nucleobases are expanded in a different direction [217]. Additional examples include nucleobase analogs tethered to the sugar moiety via alkyne linkers [218], as well as purine–pyridazine pairs modified with complementary hydrogen-bonding sites in the major groove [219].

These hydrogen-bonded UBPs have been primarily investigated for the development of novel biotechnology, particularly in terms of their compatibility with biological processes such as replication, transcription, and translation. The **iG**–**iC** base pair has been applied in a quantitative PCR method known as Plexor, which is used for the detection of target DNA [220]. A diagnostic system utilizing the **P**–**Z** base pair has also been developed for the surveillance of emerging pathogens [221]. DNA aptamers capable of binding to targets such as tumor cells have been obtained through SELEX using three types of base pairs: A–T, G–C, and **P**–**Z** [222]. Similarly, DNAzymes with efficient RNA-cleaving activity have recently been identified, in which the unnatural **Z** bases provide a general acid–base functionality [223]. Furthermore, some UBPs were found to be transcribed into RNA. Artificial DNA and RNA molecules containing eight different nucleobases or four types of base pairs (i.e., A–T, G–C, **P**–**Z**, and **B**–**S**) have been reported, enabling the enzymatic synthesis of an eight-letter version of the fluorescent Spinach RNA aptamer [211]. In addition, translation systems with **iG**–**iC** [224] or **s**–**y** base pairs [212] have been developed, allowing for the site-specific incorporation of noncanonical amino acids. For example, the **s**–**y** base pair has been shown to function in both transcription and translation, incorporating 3-chlorotyrosine corresponding to a “CT**s**” codon [212]. Since UBP systems reduce interference with natural nucleic acids, DNA and RNA containing UBPs hold great promise for broad applications in biotechnology, diagnostics, and medicine.

**Figure 11:**
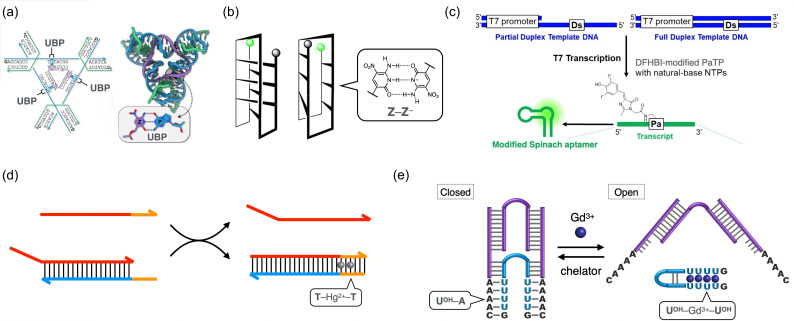
(**a**) Design and structure of tensegrity triangle DNA crystals containing unnatural **P**–**Z** base pairs. Adapted from ref. [225], CC BY 4.0. (**b**) Possible structures of the fZ-motif. Adapted from ref. [227], CC BY 4.0. (**c**) Scheme of the RNA transcription for the incorporation of the DFHBI-conjugated **Pa** nucleotide at specific positions in the Spinach RNA aptamer. Adapted with permission from ref. [238]. Copyright 2022 Wiley-VCH GmbH. (**d**) Schematic representation of ${\mathrm{Hg}}^{2+}$-triggered DNA strand displacement reactions. (**e**) Schematic representation of ${\mathrm{Gd}}^{3+}$-responsive DNA tweezers. **U** represents $U^{\mathrm{OH}}$ nucleotides.

Aiming at applications in structural DNA nanotechnology, three-dimensional DNA crystals incorporating the **P**–**Z** base pair have been constructed in recent studies [225]. Benner and Sha et al. incorporated **P**–**Z** base pairs into a well-established tensegrity triangle motif originally developed by Seeman’s group (Figure 11a) [226]. The modified DNA triangle was shown to self-assemble successfully in a manner analogous to natural DNA systems, yielding highly ordered 3D crystals. X-ray crystallographic analysis confirmed that the overall structure closely resembled that of the original DNA motif composed only of Watson–Crick base pairs. These results demonstrate that the self-assembly of DNA strands containing UBPs can also be programmed, supporting the utility of UBPs in DNA nanotechnology. In addition, a novel folding motif termed the fZ-motif has been discovered for DNA strands containing multiple **Z** bases (Figure 11b) [227]. The **Z**-rich strands were found to fold into four-stranded structures via the formation of consecutive deprotonated **Z**–$Z^{-}$ base pairs under basic conditions, expanding the repertoire of nucleic acid structures. Regarding DNA information storage, a 12-letter DNA system was developed in which four types of UBPs with different hydrogen-bonding patterns are integrated [228] Both ‘writing’ and ‘reading’ of the base sequences were achieved through enzyme-assisted synthesis and nanopore sequencing. Consequently, the incorporation of UBPs enhances both the molecular addressability and the structural diversity of DNA, which is expected to contribute to the design of sophisticated DNA nanoarchitectures.

### 4.2. Hydrophobic Artificial Base Pairs

It may be surprising that nucleobase pairing can occur without hydrogen bonding. Nevertheless, numerous examples of non-hydrogen-bonded hydrophobic unnatural base pairs (UBPs) have been reported (Figure 10d). Among them, Romesberg’s group has developed a series of such UBPs through extensive screening efforts. One representative example is the **MMO2**–**5SICS** base pair, in which the methoxy group of **MMO2** hinders its interaction with natural nucleobases, while the methyl group of **5SICS** serves to suppress the formation of an undesired **5SICS**–**5SICS** self-pair [229]. An optimized derivative, the **NaM**–**5SICS** base pair, demonstrated high efficiency and fidelity in both PCR amplification by DNA polymerase [230] and transcription by RNA polymerase [231].

Hirao’s group has developed another class of hydrophobic UBPs, exemplified by the **Ds**–**Pa** [232] and **Ds**–**Px** pairs [233], which were strategically designed based on the principle of shape complementarity. The **Pa** base with a pyrrole scaffold is structurally smaller than natural pyrimidine bases and selectively pairs with bulky counterparts such as the **s** base and its improved derivative, **Ds** [234]. These findings demonstrated that selective base pairing can be achieved through shape complementarity without relying on hydrogen bonding. In enzymatic replication experiments, γ-amidotriphosphate derivatives of **Ds** and A were employed to suppress undesired formation of **Ds**–**Ds** and A–**Pa** mispairs [232]. This strategy enabled highly efficient PCR amplification of DNA containing **Ds**–**Pa** UBPs alongside canonical A–T and G–C pairs with a per-cycle selectivity exceeding 99.9%. The **Pa** base was subsequently optimized to yield the **Px** base [233]. A nitro group was introduced to prevent A–**Px** mispairing via electrostatic repulsion, while the propargyl group was added to enhance the incorporation efficiency of **Px** opposite **Ds**. These modifications led to improved PCR performance. The shape-complementary nature of the **Ds**–**Px** pair was confirmed by X-ray crystallographic analysis of its complex with DNA polymerase [235].

These hydrophobic UBPs have been applied in a variety of biological contexts. For example, the **Ds**–**Px** base pair has been utilized in quantitative PCR. Fluorescent analogs of the **Ds** base, such as **Dss**, have been used to develop DNA detection methods similar to Plexor [236]. The hydrophobic **s**–**Pa** base pair was shown to be transcribable [234,237] and used for site-specific fluorescent labeling of RNA. The fluorescence intensity of the **s** base is sensitive to stacking interactions with neighboring bases, allowing for its use in the analysis of RNA structure and dynamics [234]. Based on **Dx**–**Pa** base pairing, various chemical modifications have been introduced into RNA through transcription using modified **Pa** triphosphate substrates. For example, a Spinach RNA aptamer covalently linked to its fluorogenic cofactor (DFHBI) were synthesized via transcription (Figure 11c) [238]. Other hydrophobic base pairs, such as **MMO2**–**5SICS**, have also been applied in RNA labeling strategies [239].

Taking advantage of the high fidelity of the **Ds**–**Px** base pair in PCR, Hirao’s group has developed SELEX methods that incoporate hydrophobic UBPs [240]. Using a DNA library containing the **Ds** bases, they successfully identified DNA aptamers that bind to target proteins such as interferon-γ (IFN-γ) and human vascular endothelial growth factor (VEGF). These **Ds**-containing aptamers exhibited remarkably high affinities toward their targets, owing to the introduction of the **Ds** base, which imparts additional chemical properties, such as hydrophobicity, to canonical DNA. SELEX technologies based on the **Ds**–**Px** base pair continue to evolve [241,242] and have been further applied to generate DNA aptamers targeting cancer cells [243], highlighting their growing potential in biomedical applications.

One of the most ambitious applications of UBPs is the creation of semi-synthetic organisms (SSOs) possessing an expanded genetic alphabet. Following extensive efforts to overcome several challenges such as the transport of unnatural nucleoside triphosphates into cells, Romesberg’s group successfully developed an SSO containing six-letter DNA using the **NaM**–**5SICS** base pair or its optimized analogs [244,245]. These UBPs were shown to be stably replicated within engineered *E. coli* cells. Moreover, in vivo transcription and translation were achieved in the SSO, enabling the biosynthesis of proteins containing noncanonical amino acids [246]. These advances in synthetic biology significantly broaden the potential of UBPs to create biomolecular systems beyond the limitations of nature.

### 4.3. Metal-Mediated Artificial Base Pairs

As an alternative pairing motif explored for unnatural base pair (UBP) systems, metal coordination bonds have been employed to construct metal-mediated artificial base pairs (Figure 10e) [247,248,249,250]. It has been known since the early 1960s that natural T bases can form an unnatural T–${\mathrm{Hg}}^{2+}$–T base pair in the presence of ${\mathrm{Hg}}^{2+}$ ions [251,252]. Later studies revealed that the T–${\mathrm{Hg}}^{2+}$–T base pair is formed through coordination of the N3 atoms of deprotonated T bases [253,254]. The association constant ($K_{a}$) for ${\mathrm{Hg}}^{2+}$ binding to a T–T mismatch inside a DNA duplex was estimated to be $5\times 10^{5}$$M^{-1}$ [255]. Similarly, natural C bases have been shown to form ${\mathrm{Ag}}^{+}$-mediated base pairs, C–${\mathrm{Ag}}^{+}$–C [256,257].

Various artificial nucleobases bearing metal-coordinating functionalities have been developed to form metal-mediated UBPs. The coordination geometries of these base pairs are generally designed to be linear or planar to allow them to stack compatibly with neighboring base pairs. For example, Shionoya’s group synthesized hydroxypyridone-type nucleobase (**H**), which forms a square-planar 2:1 complex with a ${\mathrm{Cu}}^{2+}$ ion (i.e., **H**–${\mathrm{Cu}}^{2+}$–**H** base pair) [258]. Because metal-mediated base pairs form only in the presence of the corresponding metal ions, the thermal stability of DNA duplexes can be altered by the addition of those metal ions. For instance, the melting temperature ($T_{m}$) of a 15-mer duplex containing a **H**–**H** mismatch was increased by +13.1 °C upon addition of ${\mathrm{Cu}}^{2+}$. This behavior is particularly useful for developing metal-responsive dynamic DNA systems (vide infra). A wide variety of metal-mediated UBPs have been reported utilizing transition metal ions such as ${\mathrm{Hg}}^{2+}$, ${\mathrm{Ag}}^{+}$, ${\mathrm{Cu}}^{2+}$, and ${\mathrm{Ni}}^{2+}$. Carell’s group reported a salen complex-type base pair, **S**–M(en)–**S** (M = ${\mathrm{Mn}}^{3+}$, ${\mathrm{Cu}}^{2+}$, etc.), in which the opposing salicylic aldehyde nucleobases (**S**) are crosslinked via reversible covalent bonding with ethylenediamine (en) and coordination to the metal ion [259]. Due to this additional covalent linkage, the formation of **S**–M(en)–**S** leads to extraordinary duplex stabilization ($\Delta T_{m}$ = +42.5 °C for **S**–${\mathrm{Cu}}^{2+}$(en)–**S**).

Shionoya and Takezawa et al. developed metal-responsive bifacial nucleobases that can form both hydrogen-bonded and metal-mediated base pairs depending on the presence of the specific metal ions [260,261,262]. For example, a 5-hydroxyuracil nucleobase ($U^{\mathrm{OH}}$) can form a hydrogen-bonded $U^{\mathrm{OH}}$–A base pair via its Watson–Crick face, while also forming metal-mediated homopairs such as $U^{\mathrm{OH}}$–${\mathrm{Gd}}^{3+}$–$U^{\mathrm{OH}}$ on its opposite side (Figure 10f) [260]. The 4-carbonyl and the 5-hydroxy groups serve as a bidentate ligand that coordinates to the bridging metal ions. It is noteworthy that additional ligand molecules, such as water and neighboring nucleobases, would also bind to the ${\mathrm{Gd}}^{3+}$ ions, which typically adopts an 8- or 9- coordination geometry. Similarly, a 5-carboxyuracil (**caU**) base was found to form not only a Watson–Crick-type **caU**–A base pair, but also a ${\mathrm{Cu}}^{2+}$-mediated **caU**–${\mathrm{Cu}}^{2+}$–**caU** base pair (Figure 10g) [261]. In addition, **caU** has shown to form heteroleptic metal-mediated base pairs with natural nucleobases, such as **caU**–${\mathrm{Hg}}^{2+}$–T, **caU**–${\mathrm{Ag}}^{+}$–C, and **caU**–${\mathrm{Cu}}^{2+}$–G. These bifacial nucleobases enable metal-dependent control over duplex stability. For example, a 15-mer duplex containing three $U^{\mathrm{OH}}$–$U^{\mathrm{OH}}$ mismatches exhibited significant thermal stabilization upon the addition of three equivalents of ${\mathrm{Gd}}^{3+}$ ions ($\Delta T_{m}$ = +18 °C), attributed to the formation of three $U^{\mathrm{OH}}$–${\mathrm{Gd}}^{3+}$–$U^{\mathrm{OH}}$ base pairs [260]. In contrast, a duplex containing three $U^{\mathrm{OH}}$–A pairs was destabilized by ${\mathrm{Gd}}^{3+}$ addition, possibly because the binding of ${\mathrm{Gd}}^{3+}$ ions to the $U^{\mathrm{OH}}$ caused the disruption of hydrogen bonding ($\Delta T_{m}$ = −14 °C). This bifacial base-pairing property offers a promising toolbox for the design of metal-responsive DNA systems.

The metal-mediated UBPs were first applied to construct one-dimensional arrays of homologous and heterologous metal ions within DNA duplexes [263,264,265]. This system allowed for the emergence of magnetic interactions between the assembled ${\mathrm{Cu}}^{2+}$ ions [263]. Furthermore, the electrical conductivity of DNA duplexes could be modulated through the formation of **H**–${\mathrm{Cu}}^{2+}$–**H** base pairs [266]. Although still in the early stages, the polymerase incorporation of metal-mediated base pairs has also been explored [267]. For example, a salicylaldehyde-bearing nucleotide (**S**) was incorporated opposite another **S** base on the template strand via the formation of an **S**–${\mathrm{Cu}}^{2+}$(en)–**S** base pair and was subsequently applied to PCR amplification [268]. Based on the metal-mediated base pairing, various metal-responsive DNA systems have been developed [269]. DNAzymes (catalytic DNAs) capable of cleaving target RNAs have been rendered metal-responsive by incorporating metal-mediated UBPs such as T–${\mathrm{Hg}}^{2+}$–T and **H**–${\mathrm{Cu}}^{2+}$–**H** [270,271,272,273,274,275,276,277,278]. These modified DNAzymes were designed so that the formation of metal-mediated base pairs induces the reconstruction of the catalytically active structures. Similarly, metal-responsive DNA aptamers were also constructed using the same design principle [279,280].

The application of metal-mediated UBPs in DNA nanotechnology has recently gained increasing attention. For example, pyrimidine–pyrimidine mismatch pairs were introduced into DNA three-dimensional crystals composed of tensegrity triangle motifs, and metal-mediated base pairs were formed using ${\mathrm{Ag}}^{+}$ or ${\mathrm{Hg}}^{2+}$ ions [281,282]. This stable DNA nanoarchitecture allowed for structural analysis and systematic evaluation of the formation conditions for metal-mediated UBPs. In the context of dynamic DNA nanotechnology and DNA computing, metal-ion-mediated strand displacement reactions (SDRs) have been developed. The formation of T–${\mathrm{Hg}}^{2+}$–T base pairs was used to initiate binding of the input DNA to the toehold region, thereby triggering the SDR process (Figure 11d) [283]. Similarly, ${\mathrm{Gd}}^{3+}$-triggered SDRs were demonstrated using bifacial $U^{\mathrm{OH}}$ bases, where metal-mediated destabilization of $U^{\mathrm{OH}}$–A pairs and concurrent formation of $U^{\mathrm{OH}}$–${\mathrm{Gd}}^{3+}$–$U^{\mathrm{OH}}$ base pairs drove the strand displacement [284]. As prototypes of metal-responsive DNA molecular machines, DNA tweezers and DNA walkers that operate in response to ${\mathrm{Hg}}^{2+}$ ions were constructed based on the reversible formation of T–${\mathrm{Hg}}^{2+}$–T base pairs [285,286]. Kuzuya et al. developed DNA origami pliers that can be zipped via C–${\mathrm{Ag}}^{+}$–C base pairing [287]. The $U^{\mathrm{OH}}$ base was also utilized to construct ${\mathrm{Gd}}^{3+}$-responsive DNA tweezers, which switch reversibly between open and closed conformations through the formation of $U^{\mathrm{OH}}$–A and $U^{\mathrm{OH}}$–${\mathrm{Gd}}^{3+}$–$U^{\mathrm{OH}}$ base pairs, respectively (Figure 11e) [284]. The incorporation of metal-mediated UBPs is expected not only to enhance the stability of DNA nanostructures but also to confer stimuli-responsiveness. In addition, it holds potential for the development of DNA-based nanomaterials and nanodevices that exploit the electrical, magnetic, and catalytic properties of the metal centers.

### 4.4. Xeno-Nucleic Acids (XNAs) with Unnatural Backbones and Base Pairs

**Figure 12:**
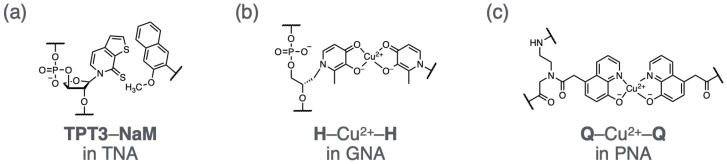
Xeno-nucleic acids (XNAs) with unnatural backbones and unnatural base pairs (UBPs). (**a**) A hydrophobic **TPT3**–**NaM** base pair incorporated within an α-L-threofuranosyl nucleic acid (TNA) duplex. (**b**) A metal-mediated **H**–${\mathrm{Cu}}^{2+}$–**H** base pair incorporated within a glycol nucleic acid (GNA) duplex. (**c**) A metal-mediated **Q**–${\mathrm{Cu}}^{2+}$–**Q** base pair incorporated within a peptide nucleic acid (PNA) duplex.

One of the important challenges in the development of ultimate nucleic acids (ΩNAs) is to synthesize DNA analogs in which both the backbone and the base pairs are engineered. Recently, a novel xeno-nucleic acid (XNA) composed of an α-L-threofuranosyl nucleic acid (TNA) backbone and hydrophobic **TPT3**–**NaM** base pairs was synthesized (Figure 12a) [288]. The TNA nucleoside triphosphates of **TPT3** and **NaM** were synthesized, and DNA-templated primer extension reactions were examined using engineered polymerases. As a result, enzymatic synthesis of a TNA strand containing a **TPT3** base was demonstrated through the formation of a **TPT3**–**NaM** base pair.

On the other hand, various examples have been reported regarding the incorporation of metal-mediated unnatural base pairs (UBPs) into noncanonical nucleic acid backbones. For instance, metal-mediated base pairs have been investigated with DNA duplexes containing α-nucleotides [289,290] as well as bridged or locked nucleic acid (BNA/LNA) backbones [291,292]. The **H**–${\mathrm{Cu}}^{2+}$–**H** base pair was formed within glycol nucleic acid (GNA) duplexes possessing an acyclic propylene glycol backbone, and its structure was elucidated by X-ray crystallography (Figure 12b) [293,294]. Due to their structural simplicity and ease of synthesis, GNA scaffolds have been regarded as useful platforms for the incorporation of metal-mediated UBPs, as demonstrated by various reports on chimeric GNA/DNA duplexes containing a single metal-mediated base pair [295,296,297]. In addition, several types of metal ligands have been introduced into peptide nucleic acid (PNA) duplexes to form interstrand 2:1 metal complexes, leading to the formation of metal-mediated base-pair analogs (Figure 12c) [298,299,300,301]. The metal-induced stabilization of PNA duplexes has been demonstrated, suggesting their potential for the development of metal-responsive PNA-based materials. Although still in their infancy, XNAs containing UBPs are expected to greatly expand the chemical diversity of nucleic acids.

## 5. Potential Applications of ΩNA

The potential applications of ΩNA span a broad range of fields, from advancing synthetic biology through the introduction of non-natural physicochemical properties and functionalities to enabling novel developments in nanobiotechnology. Especially, artificial nucleic acids and modified nucleic acids are serving as a functional tool for the construction of nanobiosystems, including DNA nanostructures, molecular computers, and their integration into molecular robots. Its properties, such as enhanced stability, faster kinetics, orthogonality to the natural nucleic acids, and the ability to incorporate non-natural functionalities, suggest and anticipate ΩNA as a promising candidate for driving progress in these emerging areas. Given that current implementations in these contexts remain limited, this section provides a perspective on future research by highlighting representative (though not exhaustive) current examples in these fields and outlining potential directions for further development.

### 5.1. DNA Nanostructures

Structural DNA nanotechnology, which makes full use of the strict sequence specificity of DNA hybridization and the uniform right-handed double helix structure of DNA, is a research field where the utilization of ΩNA is highly anticipated. Naturally occurring DNA double helices are linear strands without branching, except for temporary exceptions like the Holliday Junction observed during homologous recombination. In contrast, Seeman, the founder of structural DNA nanotechnology, proposed the “Immobile Four-way Junction” structure [10]. This structure fixes the junction site by making all four nucleotide sequences in the branches of the Holliday Junction distinct. This structure serves as the “Crossover motif”, the most fundamental element supporting this research field of structural DNA nanotechnology, used to freely connect DNA double helices. Using such branched DNA double helix structures, DNA cubes [302] and truncated octahedron [303] were initially created by employing DNA double helices as lattices. Among these, DNA tetrahedrons were found to be efficiently incorporated into cells, and are actively studied as one of the representative DDS carriers using DNA [304]. Subsequently, structural DNA nanotechnology achieved a breakthrough with the invention of the “Double Crossover (DX) motif”, which connects two DNA double helices in parallel at two crossover sites [305]. By using this motif, composed of at least five chemically synthesized DNA strands, like tiles and assembling them in an alternating pattern via sticky ends, micron-sized two-dimensional DNA sheets have been constructed [306]. This DX sheets became the first landmark example of a DNA nanostructure visualized by atomic force microscopy (AFM). Various DNA tiles were later developed, including the TX motif composed of three DNA double helices [307] and the nanomechanical PX-JX2 motif [308]. DNA two-dimensional sheets formed from these tiles are utilized as scaffolds for nanoarrays of gold nanoparticles, proteins, and other nanomaterials.

It might be widely agreed that the second breakthrough in the field of structural DNA nanotechnology was the invention of DNA origami in 2006 [309]. This technique, proposed by Rothemund, uses very long single-stranded DNA, typically M13mp18 circular phage genome, as a scaffold strand, and folds it into a single-stroke pattern using more than 200 short complementary chemically synthesized DNA strands called staple strands. It was first applied to prepare two-dimensional DNA sheet structures, but soon, three-dimensional structures such as boxes [310] or moving nanomechanical devices [287] were constructed. Moreover, the invention of a rigid “Honeycomb Lattice” design [311], that can even be bent relatively freely [312], has made it possible to create giant molecular complexes with cell sizes of up to several microns [313].

Although the field of structural DNA nanotechnology has seen highly active research involving many scientists since the 1990s, while examples of using photo-responsive units like azobenzene or ^CNV^K alone have increased, the reality is that there are very few cases where the DNA forming the structure is collectively replaced with XNA. Among these, PNA has been relatively commonly used in DNA nanostructures. The earliest example is the partial incorporation of PNA into DX tiles by Lukeman et al. in 2004 [314]. They successfully estimated the helical parameters of 7- or 8-nt PNA/DNA hybrids replaced in a DX tile by determining the base count of the hybrids that correctly formed DX tiles into two-dimensional sheets. Similarly, Gnapareddy and colleagues examined the formation of 2D DNA sheet of DX tiles or 5-helix ribbon by replacing one of the DNA components with a PNA strand [315]. Pedersen et al. later introduced PNA strands to both TX tile and DNA origami nanostructures via toehold free strand displacement [316].

In contrast to the above examples of partial replacement of PNA in DNA nanostructures, Duan et al. prepared whole PNA/PNA three-way and four-way junctions of 6-bp branches and visualized them under AFM [317]. Interestingly, results indicating that alanine residues inserted to the junction site act as a flexible spacer just like thymidine linker often used in DNA nanostructures were reported.

On the other hand, Wang et al. reported complete replacement of DNA in DX tiles with FANA [318]. FANA is known to possess high stability against enzymatic degradation and under acidic conditions, largely due to the high electronegativity of fluorine atoms, while its structural factors, such as sugar pucker, are nearly identical to those of DNA. This also applies to FANA tiles. The authors demonstrated that FANA tiles exhibit a Tm approximately 9 °C higher than DX tiles (74.7 vs. 65.5 °C) and are highly stable under acidic conditions, while FANA tiles stain similarly to DX tiles using the intercalating fluorescent dye YOYO-1.

Flory et al. prepared DNA pyramids with partial PNA/DNA portions [319,320]. They succeeded in replacing two 8-bp portions in two edges of DNA pyramids with PNA strands, and found that stepwise hybridization of PNA to DNA pyramids is possible [319]. The PNA strand was later used to attach proteins (Cytochrome C or Azurin) to DNA pyramids by preparing PNA–protein conjugates [320].

Skaanning et al. drastically employed XNA to prepare 3D DNA lattices and prepared cubes and pyramids fully replaced with L-*a*TNA duplexes quite recently in 2024 [194]. Compared to a DNA cube with a side length of 16 base pairs, which is equivalent to 1.5 turns of a canonical B-type double helix, the L-*a*TNA cube with a side length of 7 base pairs exhibited equivalent thermal stability (Tm of 63 vs. 58 °C, respectively). In contrast, while the DNA cube nearly disappeared in 6 h at 37 °C in 20% fetal bovine serum (FBS), no degradation of the L-*a*TNA cube was observed for more than 48 h. The authors further succeeded in tethering nanobodies onto the L-*a*TNA cube by utilizing triple-strand formation between poly-T and poly-A sequences.

The incorporation of multiple XNAs to DNA origami nanostructures was reported by Qin et al. in 2024 [321]. They attached TNA (Threose Nucleic Acid) to the $3^{\prime }$-end of staple strands by using terminal deoxynucleotidyl transferase (TdT) and TNA nucleoside triphosphate (tNTP) substrates. The resulting staple strands bearing one TNA residue at the $3^{\prime }$-end became intact to exonuclease digestion, adding tremendous stability toward biodegradation to DNA origami nanostructures.

LNA residues were partially incorporated into a DNA origami structure to enhance the binding between two different origami units [322]. A DNA-minimal cage designed for delivery of oligonucleotide drugs was partially modified with LNA to suppress broader melting transitions of the structures [323]. The binding arms of DNAzyme was modified with LNA, which showed improved performance due to strong binding affinity [105]. L-DNA-based molecular beacon cannot detect RNA in natural cells due to the orthogonality; however, it can be used as a smart nanothermometer free from degradation and unspecific interaction with natural D-RNAs in cells [324].

Systems utilizing PNA strands as triggers for deformation in nanomechanical DNA structures have also been reported. Yamazaki and colleagues visualized the invasion of pseudo-complementary PNA (pcPNA) into a DNA double helix using a DNA origami pinching device [325]. Similarly, Ackerman and Famulok utilized pcPNA invasion as a trigger to drive a rotaxane composed of DNA rings [326].

### 5.2. Molecular Computing

The use of ΩNA for molecular computing and the integration of such devices into molecular robots represents a particularly promising direction for future research. To date, artificial nucleic acids and modified nucleic acids have been explored in these areas by exploiting their unique properties, including:

1. Increasing stability;
2. Reducing leakage;
3. Tunable kinetics;
4. Photochemical and biological interfaces;
5. Sequence orthogonality.

Here, we highlight representative examples of molecular computing involving artificial nucleic acids and modifications of nucleic acids in a context of functionalities, with a particular focus on the potential future uses of ΩNA.

One of the most notable future applications of future ΩNA in molecular computing is the increase in stability. For instance, PNA has been proposed as a tool in DNA computing from early days of DNA computing due to its strong binding affinity to complementary DNA strands [327]. PNA-PNA interactions have been demonstrated to construct logic gates [328]. Higher stability, along with higher sensitivity in recognizing single base mutations and preventing degradation from nucleases due to unnatural backbone structure, highlights artificial nucleic acid’s potential as a robust component in molecular logic gates and circuits.

Furthermore, the recent trend and discussions on the use of enantiomers [329] could be also applied to ΩNA-based computing systems, as it has been shown that L-DNA, the nuclease resistant enantiomer (mirror image) of native D-DNA, can be used to construct logic gates and circuits that are orthogonal to natural nucleic acids [187]. Using PNA as an achiral molecule mediating the chiral molecules such as D- and L-DNA, information can be transferred between them using strand displacement reactions [188]. These examples suggest that future ΩNA-based systems could enable more reliable and durable molecular computing architectures capable of operating effectively in complex or harsh environments, including living cells [330].

Related to stability, another important characteristic of artificial nucleic acids is their ability to reduce leakage in reactions, a common challenge in DNA computing. For instance, LNA has been shown to suppress leakage in DNA strand displacement by more than 50-fold due to its enhanced binding affinity [331]. In this study, the authors reported an overall 70-fold performance improvement for DNA/LNA hybrid systems compared to all-DNA systems. LNA has also been applied to effectively repress unintended enzymatic reactions. Komiya et al. developed the L-TEAM (Low-TEmperature AMplification) system, an isothermal and leak-free method for exponential single-stranded DNA amplification [332]. The use of LNA in this system allowed the suppression of ab initio synthesis of DNA, allowing for a higher sensitivity and specificity in enzymatic DNA amplification. The use of such artificial nucleic acids can lead to more accurate and efficient molecular circuits, enhancing the overall performance of DNA-based computing systems.

Another promising direction of future ΩNA in molecular computing is the tunable reaction kinetics. For example, kinetics of toehold-mediated strand displacement reactions using acyclic xeno-nucleic acids (XNAs) have been investigated [333]. The results among three classes of acyclic XNAs, such as serinol nucleic acid (SNA), acyclic D-threoninol nucleic acid (D-*a*TNA), and acyclic L-threoninol nucleic acid (L-*a*TNA), showed that the kinetics of L-*a*TNA and D-*a*TNA were highly dependent on temperature, allowing to develop future temperature-sensitive molecular computing systems. In addition, using DNA-functionalized benzene-1,3,5-tricarboxamide (BTA) supramolecular polymers, the kinetics of strand displacement and strand exchange reactions were accelerated 100-fold due to the selective recruitment of DNA circuit components to the BTA polymer [334]. The acceleration has been successfully demonstrated by constructing multi-input AND gates, Catalytic Hairpin Assembly (CHA), and Hybridization Chain Reaction (HCR) using the polymer as a scaffold. Along with this context of introducing non-nucleic acid polymers, the use of cationic graft copolymer, poly(l-lysine)-graft-dextran (PLL-g-Dex) has been employed to accelerate the strand displacement reactions by stabilizing the DNA duplex formation [335]. Both translator-based and seesaw-gate-based circuits have been successfully accelerated by the system, showing its versatility by reducing their operation times from hours to minutes. These examples suggest that future ΩNA-based systems could enable the development of molecular computing architectures with controlled and accelerated reaction kinetics, allowing for finely tuned and faster molecular computations.

A tool for external, non-invasive control of molecular computers could represent another important direction for the future development of ΩNA. Such controllability would enable the external spatiotemporal inputs and regulation of molecular reactions, enhancing programmability and expanding the range of potential applications as artificial systems that could not be achieved only by natural nucleic acids. In this context, artificial and modified nucleic acids have been widely explored as tools to enable light-controlled reactions, offering a non-invasive and reversible means of modulation. A representative example is azobenzene-tethered DNA, which has been extensively used in molecular computing and DNA nanotechnology [65,66]. The details of the mechanism and various examples of devices utilizing photo-responsive hybridization were covered in the previous section (see Section 2.2).

In addition to photo-responsive systems that modulate hybridization, another important class of light-responsive artificial nucleic acids enables the regulation of covalent bonding between DNA strands through photocrosslinking [336] or photoligation [337]. Among these, the photocrosslinker ^CNV^K is one of the most widely used, offering ultrafast and reversible covalent bond formation [336]. Upon irradiation at 366 nm, ^CNV^K reacts within approximately 1 s to form a covalent crosslink with a pyrimidine base (C or T) positioned adjacent on the complementary strand. This reaction is fully reversible: exposure to 312 nm UV light cleaves the crosslink within roughly 60 s. Due to this rapid and wavelength-selective reversibility, ^CNV^K has been incorporated into a wide range of DNA-based devices including circuits [338], structures [339,340], and for practical applications, such as super-resolution labeling [341] and in vitro display technology [342].

Artificial nucleic acids offer a versatile approach to interfacing with and modulating biological genetic circuits as well. For instance, the photoreactive regulation of protein expression has been demonstrated using ^CNV^K, which enables site-specific photocrosslinking to target RNA [343]. In addition, $3^{\prime }$-deoxynucleotide triphosphates ($3^{\prime }$-dNTPs) are effectively used to permanently stall RNA polymerase during transcription elongation [344,345]. These examples underscore that ΩNA could provide an interface to connect and control biological systems through precisely engineered chemical approaches.

### 5.3. Molecular Robotics

One promising application area for ΩNA is molecular robotics. Molecular robotics aims to construct artificial systems by integrating various classes of molecular devices, such as sensors, processors, and actuators, to achieve autonomous and adaptive behavior from the molecular scale [12,346,347]. Incorporating artificial or chemically modified nucleic acids as core components of molecular robots can significantly expand their functionality and robustness, enabling more complex behaviors and richer interactions with surrounding environments.

Given the wide range of potential functionalities offered by ΩNA, a particularly compelling direction is to realize capabilities that are difficult or impossible to achieve using natural nucleic acids alone. In the following, we highlight representative examples of molecular robotic systems that rely on photo-responsive mechanisms enabled by artificial or modified nucleic acids, and we discuss how ΩNA could contribute to future advances in this domain.

#### 5.3.1. Amoeba-Type and Swarm Molecular Robots with Light-Gated Control

**Figure 13:**
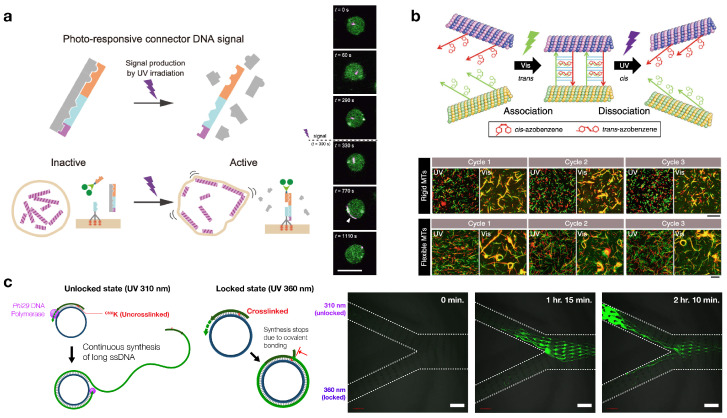
Photo-responsive molecular robots. (**a**) An amoeba-type molecular robot with light-gated activation [348]. Reprinted with permission from AAAS. (**b**) Swarm-type molecular robots with reversible light-regulated collective behaviors [349]. (**c**) Slime-type molecular robots with quasi-phototaxis behavior driven by artificial metabolism [353]. Reprinted with permission from IEEE.

Among various classes of molecular robots, amoeba-type robots provide a representative hierarchical architecture integrating sensing, processing, and actuation (Figure 13a) [348]. In this design, the processing (or signal-transduction) module functions as a YES gate, converting a light cue into mechanical output through light-gated activation of a DNA-based molecular clutch that links kinesin motors to the liposomal chassis. The photo-responsive connector DNA is initially prepared as a hairpin that covers the linker region by multiple photocleavable sites, which undergoes irreversible structural breakdown upon UV irradiation, thereby activating the linkers.

Another notable example are swarm-type molecular robots, which exhibit collective behaviors regulated by light (Figure 13b) [349]. In this system, kinesin-driven microtubules are functionalized with azobenzene-modified DNA strands. Two microtubule populations with complementary azobenzene-DNA sequences were prepared, and their light-responsive assembly is governed by the photochemical isomerization of azobenzene. Under visible light, azobenzene adopts the *trans* configuration, allowing the complementary DNA strands to hybridize and thus inducing microtubule–microtubule association, which results in swarming behavior. Upon UV irradiation, azobenzene is converted to the *cis* form, disrupting DNA hybridization, which in turn leads to disassembly of the swarm. This swarming behavior has been further exploited to achieve cooperative cargo transporting tasks [350] and localized control of swarming using light patterns [351].

#### 5.3.2. Slime-Type Molecular Robots and Prospects for ΩNA-Enabled Dynamic Functionalities

Such examples highlight the promise of artificial nucleic acids in molecular robotics, particularly for implementing light-gated control mechanisms that exceed the capabilities of natural nucleic acids. Extending this concept, future ΩNA-based systems could be incorporated into more complex chemical reaction networks, including out-of-equilibrium artificial metabolic pathways. Embedding ΩNA within such dissipative reactions would offer a route to molecular robots capable of sustained, adaptive behavior in response to environmental cues, thereby pushing molecular robotics toward more autonomous and dynamic, lifelike artificial systems powered by chemistry.

In line with this direction, slime-type molecular robots driven by artificial metabolism [352] have recently been integrated with ^CNV^K-based photochemical regulation of their metabolic reactions, enabling quasi-phototactic locomotion within microfluidic environments (Figure 13c) [353]. Here, the ^CNV^K-modified DNA strands serve as light-responsive triggers for controlling the activity of DNA synthesis reaction, which constitutes the core of the artificial metabolism powering the robot’s locomotive behavior by dynamically generating the body. Upon UV irradiation at 360 nm, the ^CNV^K moiety undergoes photocrosslinking, leading to the inhibition of DNA synthesis and consequently stopping the synthesis. On the other hand, under UV light irradiation of 310 nm, DNA synthesis is activated, allowing the robot to generate its body and move forward. The behavior has thus been regarded as a simple model of biased behavior toward specific light conditions, albeit pre-irradiation is required, to mimic phototaxis observed in living organisms. This system demonstrated how artificial nucleic acids can be used as a key component to harness dynamic, light-responsive functionalities to molecular robots operating under dissipative conditions. This emerging synergy between artificial nucleic acids and dissipative chemical processes suggests a powerful design strategy for next-generation molecular robots, in which programmable molecular components interface directly with chemomechanical processes to achieve dynamic, environment-responsive functionalities.

## 6. Discussion

### 6.1. Selection Matrix: A Design Paradigm for ΩNA

To unlock the full potential of ΩNA, it is essential to transition from a descriptive classification of artificial nucleic acids toward an application-oriented design paradigm. ΩNA should not be defined by a singular chemical structure; rather, it represents a modular and hybrid framework where various classes of artificial nucleic acid technologies are strategically integrated and tailored to meet specific technological demands. To facilitate this process, we propose a selection matrix that aligns these technologies with their functional strengths and environmental suitability (Table 2).

This table functions as a decision map where designers prioritize their requirements based on target applications. For instance, considering the requirement for high nuclease resistance in the surface region of a molecular robot intended for in vivo operation, designers might first select XNAs to construct a robust chassis. Subsequently, they can proceed with the strategic integration of functional modules, such as AMMs at the internal region, to meet specific operational goals. Such hierarchical and hybrid design logic provides an actionable framework to implement ΩNA systems.

The realization of ΩNA systems will follow a phased developmental road-map, categorized into near-, mid-, and long-term goals. First, as a near-term goal, we focus on achieving specific benchmarks, such as stability, kinetics, orthogonality, and low leakage, by optimizing individual ΩNA components for targeted functionalities. Second, as a mid-term goal, we aim to develop integrated systems capable of reconciling multiple, often conflicting, performance metrics, such as balancing high structural stability with dynamic kinetics. Finally, as a long-term goal, this trajectory will culminate in reaching the performance frontier, where ΩNA systems exhibit ultimate capabilities that may transcend the inherent limitations of natural nucleic acids. By analyzing the selection matrix, it becomes evident that a primary benchmark clarifying the gap between current implementations and future design goals to establish ΩNA ecosystem is the lack of infrastructure for scalability. To advance from lab-level proofs-of-concept to robust platforms, the development of standardized, automated, and high-throughput pipelines, including ΩNA-compatible scalable synthesis (e.g., parallel and enzymatic synthesis) and readout methods (e.g., nanopore-based sequencing or chemical decoding), is required. For instance, recent advances in nucleic acid therapeutics [6,354], in line with mRNA therapeutics [355], may lead to significant cost reductions in solid-phase nucleic acid synthesis, which can also benefit ΩNA ecosystem. In addition, addressing Ethical, Legal, and Social Issues (ELSI) [356], including the consideration of interoperability with natural nucleic acids and biological containment through extreme orthogonality, is essential for the responsible integration of ΩNA into real-world applications. Spanning from molecular synthesis to biosafety protocols, establishing a fundamental understanding of systematic approaches to integrate multiple ΩNA components in a responsible manner will represent an untapped frontier in nucleic acid chemistry.

### 6.2. Future Perspectives

The future of ΩNA holds significant promise in various fields (Table 3). First, from molecular computing to molecular robotics, the unique capabilities of ΩNA could accelerate efforts to bridge biological and synthetic molecules, enabling more robust and functional hybrid molecular systems. A key direction is the integration of ΩNA into chemical reaction networks, such as artificial metabolism and artificial central dogma, including regulatory controllers and reactive species. Such an integration would allow for programmable modulation of reaction pathways that are difficult or impossible to achieve with natural nucleic acids alone. These networks could form the basis for dynamically controlled, reactive, and interactive signal processing, autonomous feedback control, and multi-layered reactive information processing implemented entirely at the molecular scale, ultimately leading to the realization of Chemical AI and molecular cybernetics [357,358].

Beyond molecular robotics, ΩNA may offer transformative advantages in other artificial molecular systems, including DNA data storage [359]. Its enhanced chemical stability, orthogonality to natural nucleic acids, and expanded functional space allow the construction of high-density, low-error, and long-term information carriers. For example, ΩNA-mediated orthogonality would enable multi-channel storage, in which natural nucleic acids and ΩNA sequences coexist without cross-interference, thereby increasing logical capacity and permitting selective access.

Moreover, ΩNA-based systems possess the intrinsic potential to move beyond the classical DNA computing paradigm limited by Watson–Crick basepairing. By leveraging artificial base pairs, multi-state photo-responsive units, metal-mediated coordination, and noncanonical secondary structures (e.g., triplex DNA, G-quadruplexes, and i-motifs) [227], ΩNA naturally supports non-binary, multilevel information encoding. Notably, the integration of these additional higher-order structures, further stabilized and functionalized through ΩNA chemistry, offers orthogonal structural and mechanical design spaces, such as triplex-based DNA origami [360] and multi-state regulatory circuits. Such architectures could be conceptualized as quantum-inspired information units, analogous to qubits, where a single molecular element occupies multiple addressable states rather than a simple binary configuration. This architectural paradigm shift allows ΩNA to transition from traditional Boolean logic gates toward a high-dimensional computing frontier, enabling more sophisticated processing within a minimized molecular footprint.

A further speculative yet intriguing application is in the context of “mirror-life” [329], hypothetical synthetic biological systems that could evade natural immune responses and predation mechanisms. The “mirror-life” fundamentally utilizes L-DNA as genetic information, expressing life functions through transcription into L-RNA and translation into D-amino acid-based proteins. These mirror-image biopolymers are orthogonal to natural D-DNA, D-RNA, and L-amino acid-based proteins, preventing interaction with each other. The orthogonality between L-nucleic acids and D-nucleic acids is derived from differences in helical winding direction. ΩNA can be designed to create variants that interact with left-handed L-nucleic acids, right-handed D-nucleic acids, or both, enabling information mediation. Thus, ΩNA-based molecular robots could function as biochemical countermeasures against such mirror-life threats by providing a compatible synthetic interface: responsive to cues from natural nucleic acids yet capable of targeting invaders in the mirror domain. In addition to mitigating potential biosecurity risks, from a more constructive viewpoint, ΩNA’s well-controlled orthogonality creates a biologically insulated chemical space in which fully artificial molecular systems can be developed and operated with reduced concerns over unintended interactions with natural biological systems. Such an orthogonal domain could be exploited to build safer autonomous molecular robots, dynamic materials, and synthetic cellular systems that are functionally rich yet well-isolated from natural biological systems.

ΩNA is essential for research on the origin of life as well. Although the RNA world [26] is widely thought to be an early stage in the origin of life, prebiotic synthesis of the RNA monomer and polymer has still not been proven mainly due to complex stereochemistry of the cyclic ribose scaffold. As a precursor to the RNA world, there may have been a pre-RNA world in which XNA possessed genetic and catalytic functions [361]. The construction of a primitive life system using ΩNA would deepen understanding of the origin of life. In this context, ΩNA holds the potential to be the primordial nucleic acid, “Alpha Nucleic Acids (αNA).”

## 7. Conclusions

Taken together, ΩNA opens pathways to heterogeneous and hybrid molecular system architectures in which biomolecules and synthetic molecules coexist. Viewing from this system’s perspective, the unique properties of ΩNA, such as improved kinetics, enhanced stability, orthogonality, and fast and precise external controllability, would serve as a powerful design toolbox to create the next generation of molecular systems that may surpass the capabilities of natural biological systems alone. Overall, these prospects highlight the potential transformative impact of ΩNA across a wide range of scientific and technological domains, paving the way for innovative new applications of artificial molecular systems and towards a deeper understanding of life.

## Figures and Tables

**Table 2:** Example of selection matrix for ΩNA design. (AMMs: artificial molecular machine hybrids, XNAs: xeno-nucleic acids, UBPs: unnatural base pairs).

Class	Thermal Stability	Nuclease Resistance	Orthogonality	Dynamic Functionality	Cost/Scalability
Natural DNA/RNA	Baseline	Low	Low	Limited	Excellent
AMMs	High	Moderate-High	High	Very High	Low (High cost)
XNAs	Very High	High	Variable	Moderate	Moderate
UBPs	Variable	Moderate	Excellent	Moderate	Low (High cost)

**Table 3:** Possible application fields (Chemical Biology overlaps with all of these research fields, either partially or entirely) and current adoption level (Phase I: Conceptual, II: Emerging, III: Adopting, IV: Mature) of ΩNA. See Table 2 for Class.

Class	Biotechnology, i.e., PCR, Cloning	Analytical Chemistry, i.e., Aptamers	Nucleic Acid Therapeutics	Molecular Computing	Structural DNA Nanotechnology	DNA Data Storage	Synthetic Biology	Molecular Robotics, Molecular Cybernetics
Natural DNA/RNA	IV	IV	III	IV	IV	III	IV	III
AMMs	II	I	I	III	III	I	II	III
XNAs	I	II	IV	II	II	II	I	I
UBPs	III	III	I	I	I	I	II	I

## Data Availability

No new data were created or analyzed in this study.
